# Perineural invasion in intrahepatic cholangiocarcinoma: Meta-analysis of prevalence, association with tumor features, and impact on survival

**DOI:** 10.1016/j.jhepr.2026.101939

**Published:** 2026-06-25

**Authors:** Johannes Kolck, Timo Alexander Auer, Camilla Winther Pedersen, Clarissa Hosse, Jana Ihlow, Felix Krenzien, Wenzel Schöning, Uli Fehrenbach, Dominik Geisel

**Affiliations:** 1 Charité – Universitätsmedizin Berlin, Department of Radiology, Berlin, Germany; 2 Caritas Klinik – Pankow, Department of Gastroenterology, Berlin, Germany; 3 Charité – Universitätsmedizin Berlin, Department of Pathology, Berlin, Germany; 4 Charité – Universitätsmedizin Berlin, Department of Surgery CCM / CVK, Berlin, Germany

**Keywords:** Biliary tract cancer, Hepatobiliary malignancy, Liver malignancy, Nerve sheath invasion, Tumor biology, Subtypes, Mass-forming, Periductal-infiltrating, Intraductal growth, Large-duct type, Small-duct type, Biomarker, Prevalence, Curative resection, Outcome, Prognosis, Systematic review, PRISMA, Node-negative disease, Hazard ratio

## Abstract

**Background & Aims:**

Perineural invasion (PNI) is a recognized marker of tumor aggressiveness in several malignancies, but its prevalence and prognostic significance in intrahepatic cholangiocarcinoma (ICC) remain uncertain. This systematic review and meta-analysis aimed to estimate the pooled prevalence of PNI and evaluate its association with overall survival (OS) in ICC.

**Methods:**

Following PRISMA guidelines, PubMed, Web of Science, and the Cochrane Library were searched through August 2025. The PNI prevalence was pooled using a random-effects model. Where available, multivariable hazard ratios for OS were pooled using a random-effects meta-analysis. Heterogeneity was assessed and potential moderators were explored using meta-regression. Pairwise ecological correlations between PNI and other tumor features were analyzed using Pearson’s correlation coefficient.

**Results:**

We analyzed 61 studies including 15,502 patients. The pooled prevalence of PNI was 36.0% (95% CI 31.0–41.0%), with substantial heterogeneity (*I*^2^ = 98.5%). Meta-regression identified publication year and study size as independent moderators, with more recent and larger studies reporting lower PNI prevalence. PNI positively correlated with lymph node metastasis, lymphovascular invasion, and vascular invasion (all *p* <0.001), and inversely correlated with the proportion of mass-forming ICC (*p* = 0.025). Of 44 studies assessing OS, 23 were included in meta-analysis, demonstrating that PNI was associated with worse survival (hazard ratio 1.79, 95% CI 1.38–2.19, *p* <0.001).

**Conclusions:**

PNI occurs in approximately one-third of patients with ICC, is associated with poorer outcomes, and clusters with lymph node metastasis and vascular invasion, reflecting aggressive tumor biology. Despite the heterogeneity of the available data, its adverse prognostic impact is consistent.

**Impact and implications:**

Perineural invasion (PNI) is a proposed marker of tumor aggressiveness in intrahepatic cholangiocarcinoma (ICC); however, its prevalence and prognostic significance have remained uncertain, warranting systematic evaluation. This study provides the most comprehensive synthesis to date of PNI in ICC demonstrating that PNI occurs in approximately one-third of ICC cases and clusters with adverse pathological features, although its independent impact on survival remains debatable. These findings are relevant for clinicians and researchers involved in ICC management, as they suggest that PNI may contribute to more refined risk stratification, particularly in early-stage disease. In practice, PNI assessment could support clinical decision-making and patient counseling. However, future standardized and prospective studies are needed to clarify its prognostic value and potential role in guiding treatment strategies.

## Highlights

• Perineural invasion (PNI) occurs in approximately one-third of patients with resected intrahepatic cholangiocarcinoma.
• PNI clusters with adverse tumor features, such as lymph node metastasis and vascular invasion, and is associated with adverse survival outcomes.
• These findings suggest that PNI may reflect aggressive tumor biology rather than act as an independent prognostic factor.

## Graphical abstract

**Figure:**
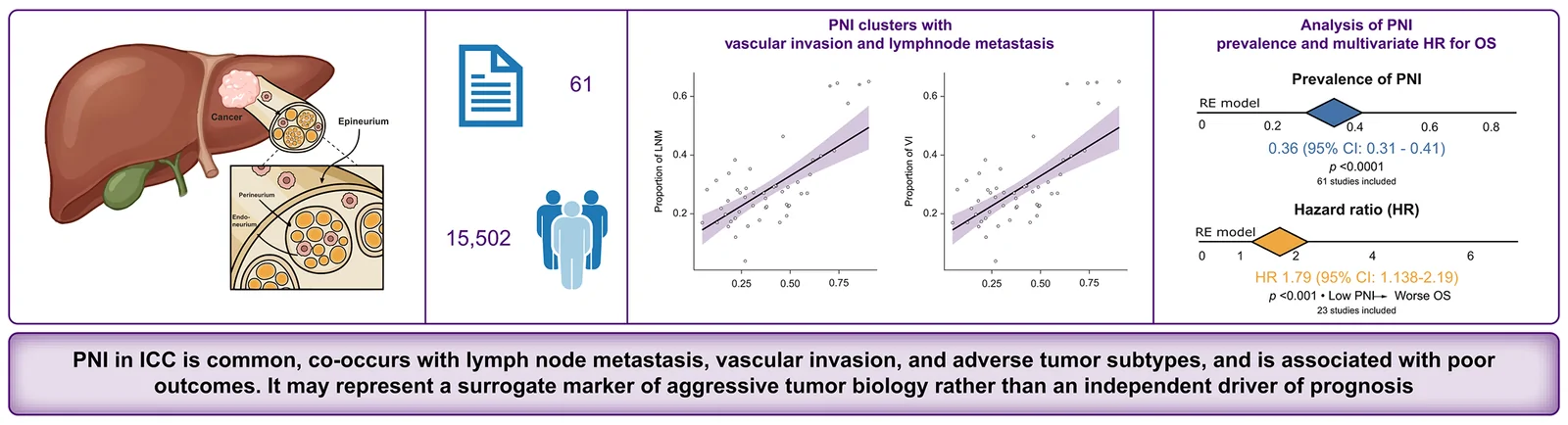

## Introduction

Intrahepatic cholangiocarcinoma (ICC) is the second most common primary hepatic malignancy after hepatocellular carcinoma, and its incidence has been steadily increasing worldwide over the past decades.^1^ ICC typically arises from second-order bile ducts, shows aggressive behavior, and often remains asymptomatic until advanced stages.^2^^,^^3^ Three main types of ICC are recognized: (1) mass-forming (MF), which appears as a distinct mass in the liver parenchyma; (2) periductal-infiltrating (PI), which spreads longitudinally along the bile duct, often causing upstream dilatation; and (3) intraductal growth (IG), which grows papillarily into the duct lumen, sometimes resembling a tumor thrombus.^4^ At diagnosis, up to 54% of patients have unresectable tumors, whereas only ∼35% qualify for surgery, the mainstay of curative intended therapies.[5], [6], [7] Because of the lack of effective treatment and the high rate of recurrence (57.9–73.4%), the prognosis after resection in these patients is dismal, with a 5-yr survival rate of only 20–40%.^8^^,^^9^

Prognostic stratification in ICC is therefore of critical clinical importance. Beyond established factors such as tumor size, vascular invasion (VI), and lymph node metastasis (LNM), perineural invasion (PNI) has increasingly been recognized as a potential histopathological marker of tumor aggressiveness.[10], [11], [12]

**Fig. 1:**
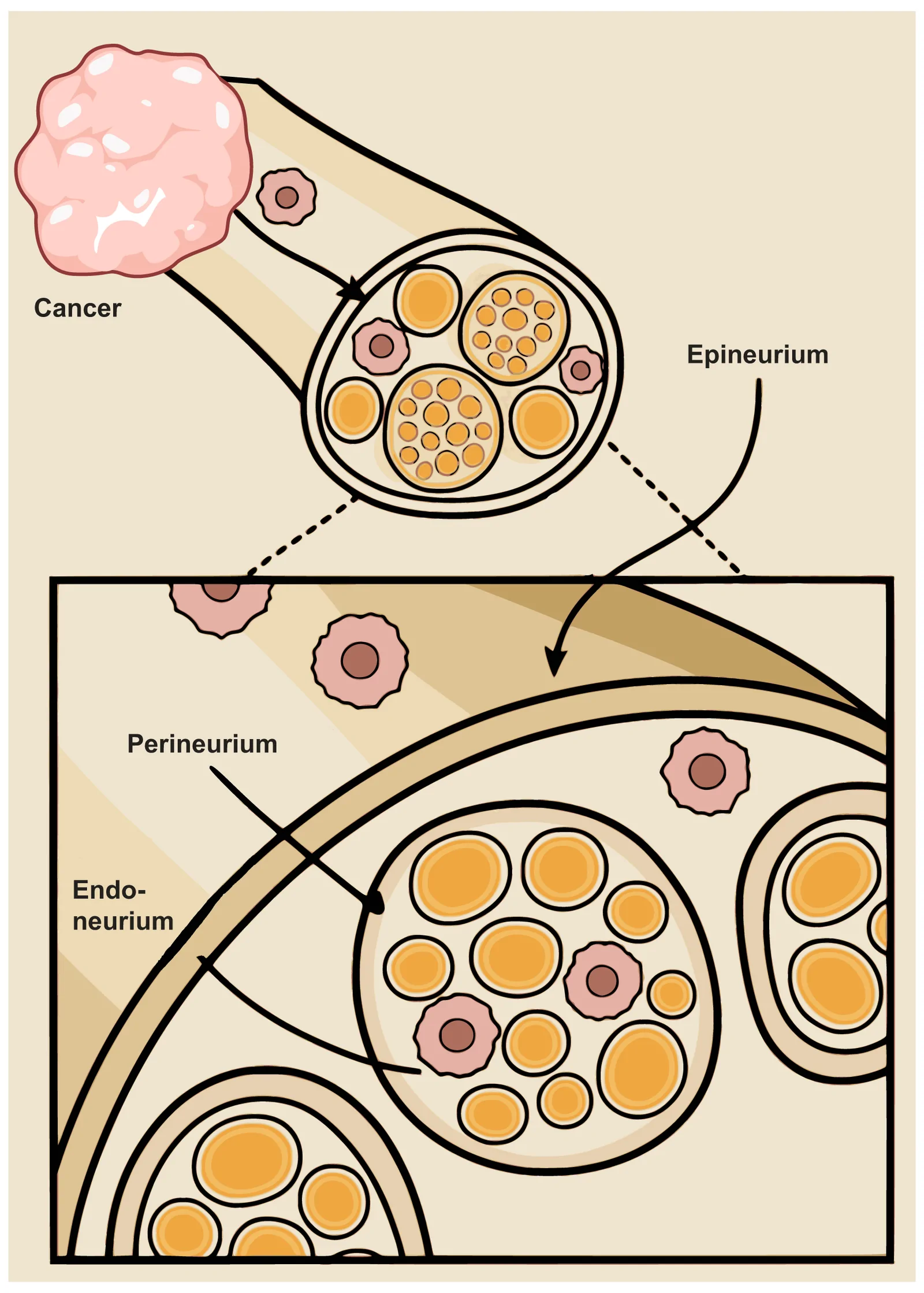
Proportional forest plot illustrating the prevalence of perineural invasion in intrahepatic cholangiocarcinoma. Across 61 studies, the pooled prevalence was 36.0% (95% CI 31.0–41.0%) using a random-effects model. Substantial between-study heterogeneity was observed (Cochran's Q = 2943.41, df = 60, *p* <0.01; I² = 98.47%; τ² = 0.182), supporting the use of the random-effects model. PNI, perineural invasion. Adapted from Bahmad *et al.*[^84^]

PNI is defined as the invasion of tumor cells into the perineural space, representing a distinct route of tumor dissemination (Fig. 1) compared with vascular or lymphatic spread.^13^

In extrahepatic cholangiocarcinoma (ECC), the presence of PNI has consistently been associated with poor prognosis.^14^^,^^15^ In ICC, however, the prognostic impact of PNI is less clear, with studies reporting widely variable prevalence rates and inconsistent associations with overall survival (OS).^16^

Given these discrepancies, a systematic synthesis of the available evidence is warranted. Therefore, we conducted the present meta-analysis with two main aims: (1) to define the pooled prevalence of PNI in ICC, and (2) to investigate the association between PNI and OS in patients with ICC.

## Materials and methods

### Search strategy and study selection

We conducted this systematic review and meta-analysis in accordance with the Preferred Reporting Items for Systematic Reviews and Meta-Analyses (PRISMA) guidelines.^17^ The protocol was preregistered on Open Science Framework Registries before data extraction/analysis (DOI 10.17605/OSF.IO/YN4UB, 05 September 2025). A comprehensive literature search was performed in PubMed, Web of Science, and the Cochrane Library up to 31 August 2025. The search strategy combined controlled vocabulary terms, where available (*e.g.* MeSH terms in PubMed), with free-text keywords and their combinations, including ‘intrahepatic cholangiocarcinoma’, ‘intrahepatic bile duct cancer’, ‘ICC’, ‘perineural invasion’, and ‘PNI’. Only articles in English language were selected.

Studies were included if they met the following criteria: (1) patients were diagnosed with ICC (histologically confirmed); (2) PNI was assessed histopathologically; and (3) the prevalence of PNI was reported.

Exclusion criteria comprised case reports, reviews, conference abstracts, studies with fewer than 20 patients, and those not differentiating ICC from other cholangiocarcinoma subtypes.

### Data extraction and quality assessment

Two reviewers (CWP and JK) independently screened titles and abstracts, assessed full texts, and extracted data, with discrepancies resolved by a third reviewer (CH). Extracted variables included study characteristics (first author, year, country, design, and cohort size), patient and tumor features (age, sex, tumor size, PNI status, LNM, lymphovascular [LVI], and VI), and survival outcomes. Study quality was assessed using the JBI critical appraisal tool for the assessment of risk of bias for cohort studies.^18^ To assess the impact of PNI on OS, studies were categorized into three groups: (1) PNI not included in OS analysis or OS not analyzed; (2) OS analyzed but PNI not significant; and (3) OS analyzed and PNI significant.

### Statistical analysis

The pooled prevalence of PNI across studies was estimated using both fixed- and random-effects models. Owing to substantial between-study variability, the random-effects model was selected as the primary approach. Proportions were logit-transformed before pooling. Between-study heterogeneity was assessed using Cochran’s Q statistic, the *I*^2^ statistic, and τ^2^. An *I*^2^ value >50% was considered indicative of substantial heterogeneity. To assess robustness, leave-one-out sensitivity analyses were performed in which each study was sequentially excluded and the pooled prevalence recalculated.

To evaluate the role of PNI as a prognostic factor for OS, multivariable hazard ratios (HRs) were extracted from the original articles, and a random-effects meta-analysis was performed. When not directly reported, SEs were derived from the corresponding 95% CIs using established methods described in the Cochrane Handbook for Systematic Reviews of Interventions. Moderator analyses were performed using univariable and multivariable random-effects meta-regression models.

The association of PNI with other tumor characteristics was assessed using study-level prevalence of PNI, LNM, VI, poor differentiation (≥G3), and the proportion of MF tumors, which were extracted from each included study. Pairwise ecological correlations between these variables were evaluated using Pearson’s correlation coefficient. Analyses were restricted to studies with available data. Studies reporting only a single subtype of ICC were excluded from the correlation analysis. All analyses were conducted using R version 4.4.2 (R Foundation for Statistical Computing, Vienna, Austria), with *p* values <0.05 considered statistically significant.

## Results

### Study cohort

**Fig. 2:**
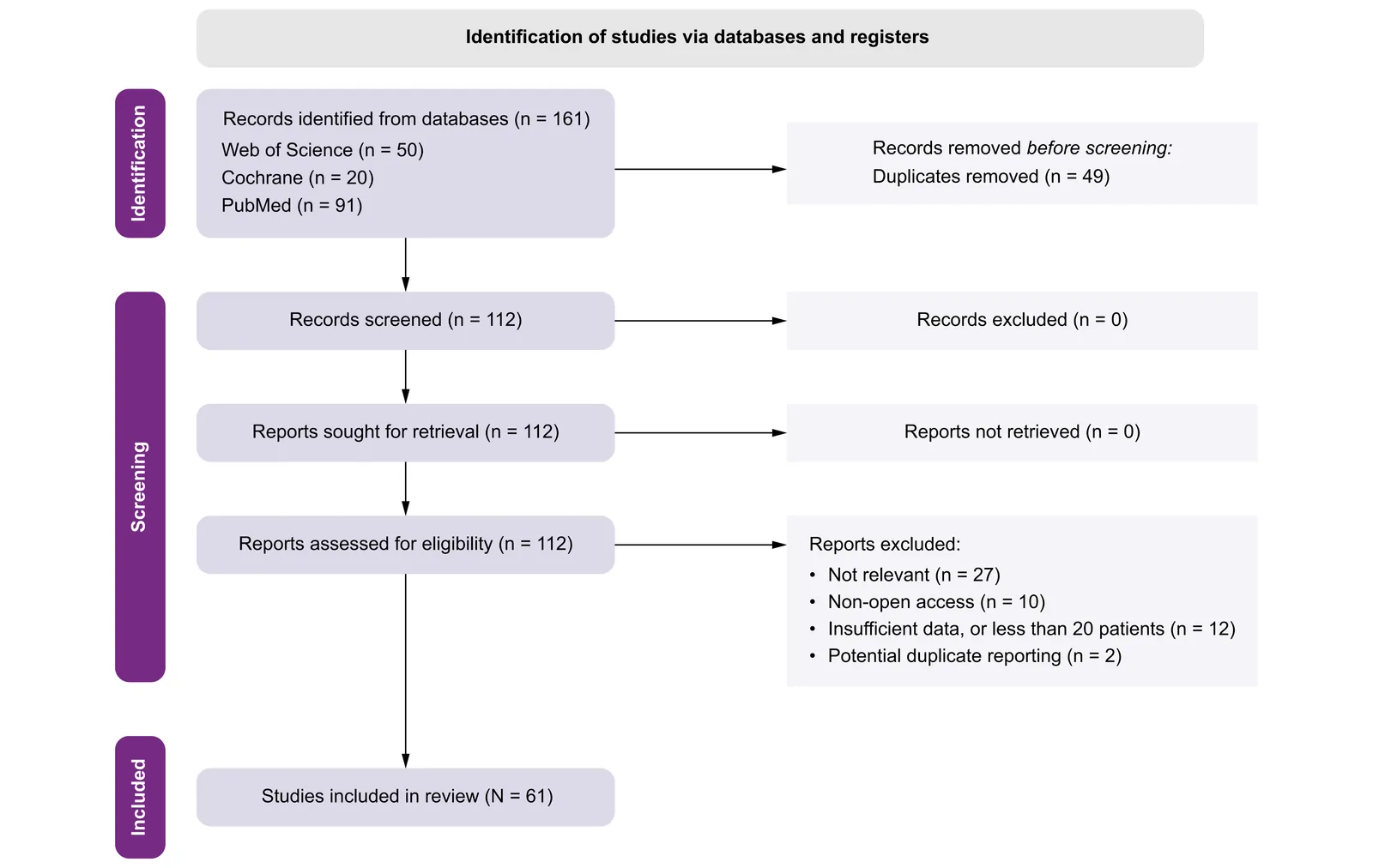
Flow diagram illustrating the selection process of studies included in the meta-analysis.

A total of 61 retrospective studies with 15,502 patients were included in the meta-analysis.^16^^,^[19], [20], [21], [22], [23], [24], [25], [26], [27], [28], [29], [30], [31], [32], [33], [34], [35], [36], [37], [38], [39], [40], [41], [42], [43], [44], [45], [46], [47], [48], [49], [50], [51], [52], [53], [54], [55], [56], [57], [58], [59], [60], [61], [62], [63], [64], [65], [66], [67], [68], [69], [70], [71], [72], [73], [74], [75], [76], [77], [78], [79] The study selection process is illustrated by the PRISMA flow chart (Fig. 2). The median cohort size was 103 patients (range 20–1,728). The mean age reported in 48 studies was 62.0 yr (SD 2.9; range 54–68 yr). Sex distribution was available in 57 studies. Overall, 59% of patients were male and 41% were female. The majority of studies was based on patient cohorts in Asia (72.13%). Tumor size and further clinicopathological characteristics (*e.g*. LNM, VI) are reported in Table 1. A minority of studies indicated the application of adjuvant therapy, whereas neoadjuvant treatment was not reported. Risk-of-bias analysis revealed that nearly all included studies fulfilled the critical domains relevant to the primary outcome, indicating that the pooled prevalence estimate was derived predominantly from methodologically valid data (Table S1).

**Table 1:** Descriptive statistics of included studies.

	Mean	SD	Minimum	Maximum	Missing
No pts	254.13	361.10	20.00	1,728.00	0
PNI (%)	36.4	18.6	4.9	90.4	0
Age (years)	62.01	2.94	54.00	68.00	13
Male (%)	56.7	8.2	34.5	72.3	4
Tumor size (cm)	5.71	1.02	4.00	8.00	23
MF (%)	72.5	16.4	35.5	95.8	38
PD (%)	29.9	18.4	2.6	81.8	9
LNM (%)	27.9	11.8	3.8	65.1	12
VI (%)	41.9	22.8	5.3	97.6	28
LVI (%)	46.3	21.7	13.1	97.1	32
Adjuvant chemotherapy (%)	33.2	10.6	12.7	66.0	42
Publication year	2,017.2	6.1	2,002.0	2,025.0	0

The prevalence of certain features, including adjuvant chemotherapy, was reported in only a limited number of studies and is therefore of limited generalizability. LNM, lymph node metastasis; LVI, lymphovascular invasion; MF, mass-forming ICC subtype; PD, poor differentiation; PNI, perineural invasion, VI, vascular invasion.

### Prevalence of PNI

**Fig. 3:**
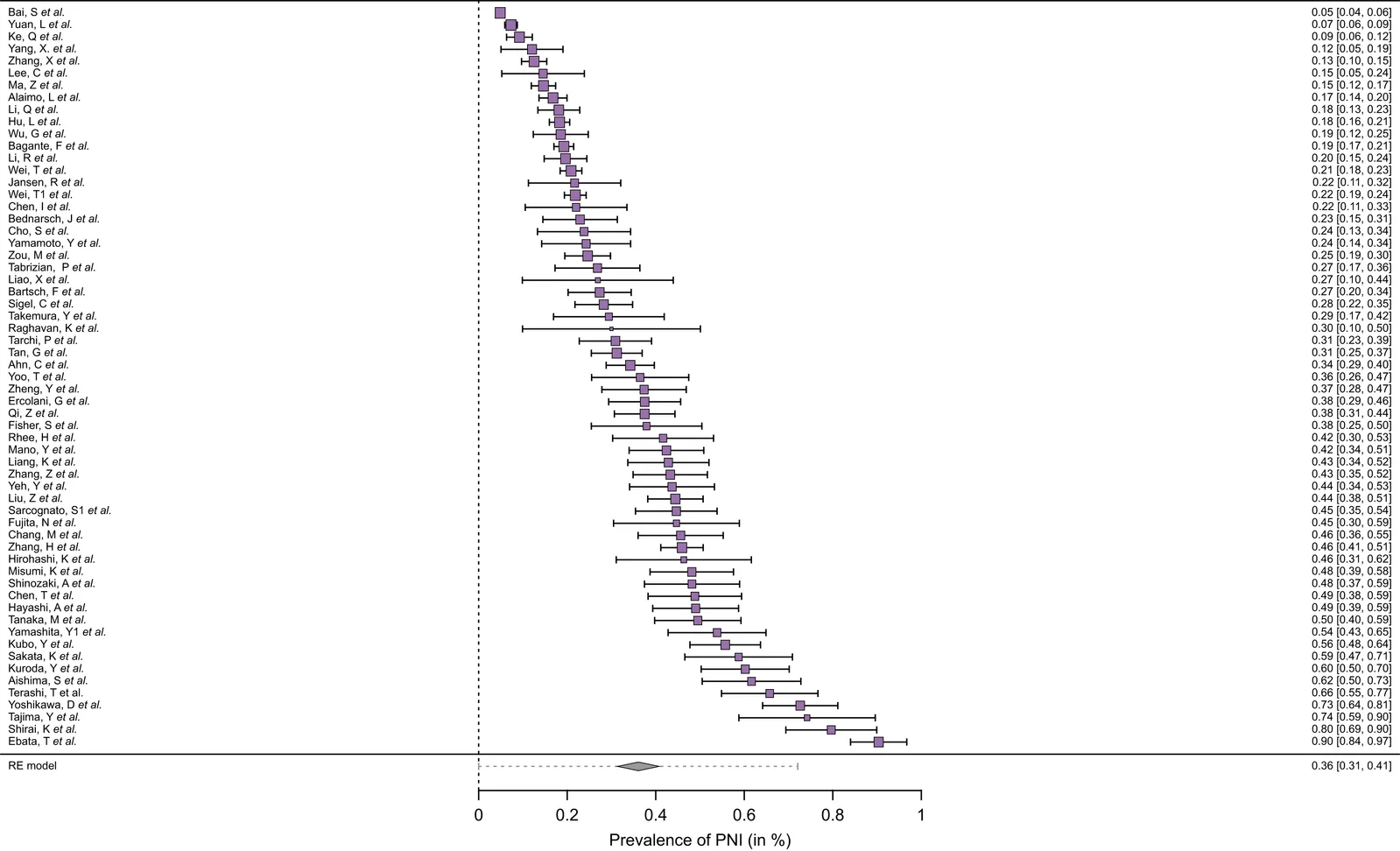
Proportional forest plot illustrating the prevalence of perineural invasion in intrahepatic cholangiocarcinoma. PNI, perineural invasion.

Across 61 studies, the pooled prevalence of PNI was 36.0% (95% CI 31.0–41.0%) under a random-effects model. Cochran’s Q = 2943.41 (degrees of freedom [df] = 60, *p* <0.01), *I*^2^ = 98.47%, and τ^2^ = 0.182, indicating high between-study heterogeneity and supporting the use of random-effects models (Fig. 3). Sensitivity analysis implementing a leave-one-out approach demonstrated that the pooled prevalence of PNI was highly robust. Excluding each study in turn yielded pooled prevalence estimates ranging from 35.1% to 36.6% compared with the overall estimate of 36.0% (95% CI 31.0–41.0%). This indicates that no single study exerted a disproportionate influence on the summary effect. Assessment of small-study effects showed a large Rosenthal fail-safe N (123,014), suggesting that the pooled result would require many null studies to lose statistical significance. However, both the rank correlation test (Kendall’s tau = 0.201, *p* = 0.022) and Egger’s regression test (Z = 4.344, *p* <0.001) were significant, indicating statistical evidence of funnel plot asymmetry and possible small-study effects. Because funnel plot asymmetry is not specific to publication bias, these findings should be interpreted cautiously.

### Moderator analysis (PNI prevalence)

Univariable random-effects meta-regressions (Paule-Mandel τ^2^, Knapp-Hartung inference) identified several significant continuous moderators of PNI prevalence. Publication year was strongly associated with lower reported prevalence (β = -0.118, *p* <0.001), explaining ∼35% of between-study heterogeneity. Similarly, larger study size (log-transformed N) predicted lower PNI prevalence (β = -0.092, *p* <0.001), accounting for ∼27% of heterogeneity. Mean cohort age showed a modest positive association (β = +0.099, *p* = 0.015), but explained only ∼11% of heterogeneity. The cohort location (Asia, USA, Europe) was not a significant moderator.

**Fig. 4:**
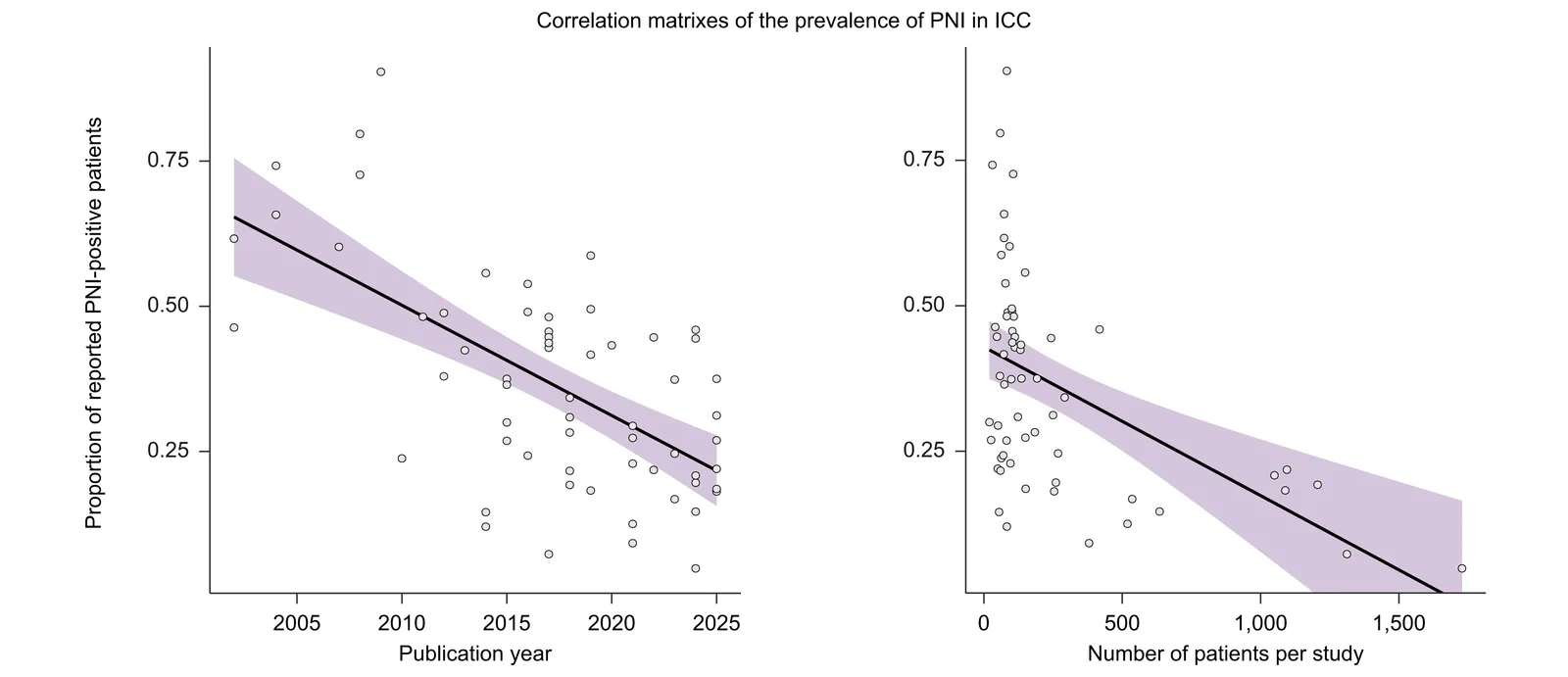
**Correlation matrixes illustrating the two identified sources of heterogeneity (publication year;*p* <0.001) and studysize;*p* = 0.014) in the prevalence of PNI in ICC**. ICC, intrahepatic cholangiocarcinoma; PNI, perineural invasion.

In the multivariable model including publication year, study size, and age simultaneously, only publication year (β = –0.084, *p* <0.001) and study size (β = -0.057, *p* = 0.014) remained significant independent predictors of PNI prevalence (Table 2), together explaining ∼46% of between-study heterogeneity. Age did not retain significance after adjustment. The correlation of PNI prevalence with publication year and study size is illustrated in Fig. 4.

**Table 2:** Results of univariable and multivariable random-effects meta-regression analyses exploring sources of heterogeneity. The multivariable model included all three covariates simultaneously. Publication year and study size remained significant independent predictors of PNI prevalence, whereas age was not independently associated after adjustment. Bold *p* values indicate statistically significant associations (*p* <0.05).

Moderator	k	Univariate				Multivariate			
		Coefficient	SE	z	*p* value	Coefficient	SE	z	*p* value
Publication year	61	-0.118	0.019	-6.121	**<0.001**	-0.084	0.021	-3.995	**<0.001**
Study size	61	-0.092	0.020	-4.487	**<0.001**	-0.058	0.023	-2.465	**0.014**
Region	61	0.217	0.090	2.409	0.076	0.016	0.024	0.658	0.511
Patient age	48	0.054	0.025	2.113	**0.035**	0.051	0.083	0.613	0.540

### Correlation of PNI with other features

Study-level correlation analysis demonstrated that PNI prevalence was significantly positively correlated with LNM (r = 0.671, *p* <0.001), VI (r = 0.548, *p* <0.001), and LVI (r = 0.671, *p* <0.001). LNM prevalence was also significantly correlated with LVI (r = 0.482, *p* = 0.013), but not with VI (r = 0.212, *p* = 0.261). The correlation between VI and LVI did not reach statistical significance (r = 0.377, *p* = 0.150). Poor differentiation was not significantly correlated with PNI (r = 0.075, *p* = 0.599), LNM (r = −0.113, *p* = 0.470), VI (r = 0.174, *p* = 0.342), or LVI (r = −0.090, *p* = 0.660).

**Fig. 5:**
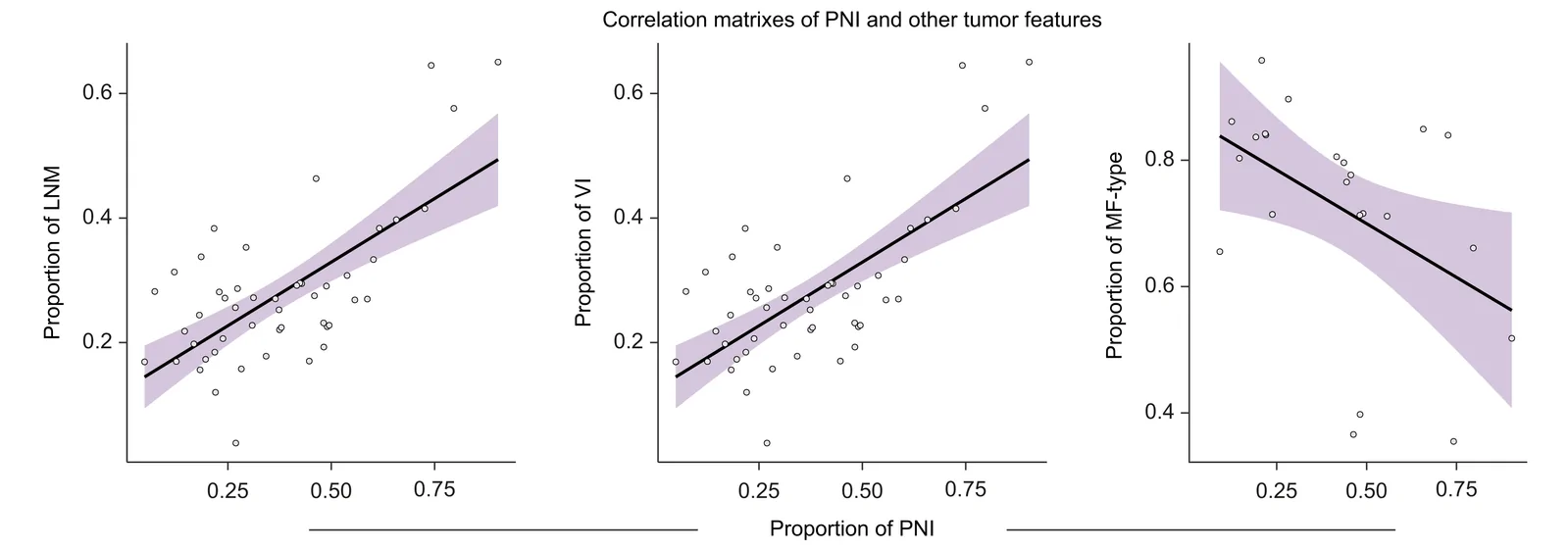
Study-level correlation analysis demonstrating that PNI prevalence was significantly positively correlated with LNM (*p* <0.001) and VI (*p* <0.001) and inversely correlated with the proportion of MF subtype (*p* = 0.025). LNM, lymph node metastasis; MF, mass-forming; PNI, perineural invasion; VI, vascular invasion.

Conversely, the proportion of the MF subtype was inversely correlated with PNI (r = −0.467, *p* = 0.025) and LNM (r = −0.563, *p* = 0.023), but showed no statistically significant correlation with VI (r = −0.342, *p* = 0.334), LVI (r = −0.380, *p* = 0.200), and poor differentiation (r = 0.187, *p* = 0.405) (Fig. 5 and Table 3).

**Table 3:** Correlation matrix of PNI with other tumor features.

		PNI (%)	LNM (%)	VI (%)	LVI (%)	PD (%)	MF (%)
PNI (%)	Pearson's r	—					
	df	—					
	*p* value	—					
LNM (%)	Pearson's r	0.671∗∗∗	—				
	df	47	—				
	*p* value	<0.001	—				
VI (%)	Pearson's r	0.548∗∗∗	0.212	—			
	df	31	28	—			
	*p* value	<0.001	0.261	—			
LVI (%)	Pearson's r	0.671∗∗∗	0.482∗	0.377	—		
	df	27	24	14	—		
	*p* value	<0.001	0.013	0.150	—		
PD (%)	Pearson's r	0.075	-0.113	0.174	-0.090	—	
	df	50	41	30	24	—	
	*p* value	0.599	0.470	0.342	0.660	—	
MF (%)	Pearson's r	-0.467∗	-0.563∗	-0.342	-0.380	0.187	—
	df	21	14	8	11	20	—
	*p* value	0.025	0.023	0.334	0.200	0.405	—

Note: ∗*p* <0.05, ∗∗*p* <0.01, and ∗∗∗*p* <0.001. LNM, lymph node metastasis; LVI, lymphovascular invasion; MF, mass-forming ICC subtype; PD, poor differentiation; PNI, perineural invasion, VI, vascular invasion.

### Impact on overall survival

**Fig. 6:**
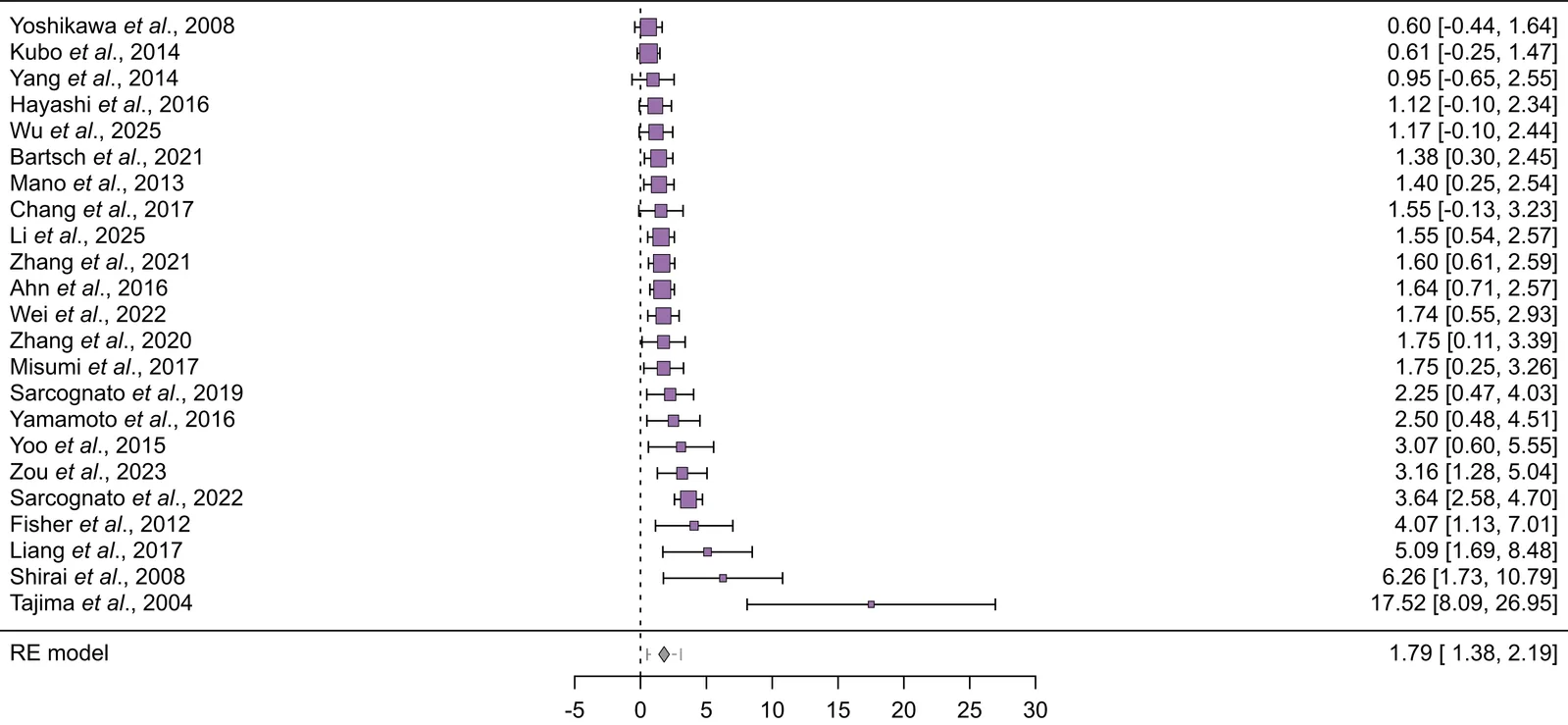
Forest plot of multivariable hazard ratios for overall survival. A random-effects model including 23 studies demonstrated a significant overall effect (estimate = 1.79, SE = 0.206, Z = 8.67, *p* <0.001), with a 95% CI ranging from 1.383 to 2.190.

Out of 44 studies assessing OS, 23 provided sufficient data for inclusion in the meta-analysis; of these 23, 15 studies comprising 4,111 patients reported PNI as significant for OS in multivariate analysis. Using a random-effects model, the presence of PNI was significantly associated with worse OS. The pooled HR was 1.79 (95% CI 1.38–2.19, *p* <0.001), indicating that patients with PNI had a significantly higher hazard of death compared with those without PNI (Fig. 6).

Several studies investigated the molecular and biological correlates of PNI, examining how tumor genetics, phenotypic markers, and growth patterns relate to its prognostic significance. Liang *et al.*^44^ examined the GALNT14 genotype and found PNI to be strongly associated with reduced OS independently of molecular subtype, periductal invasion, and mixed histology. Sarcognato *et al.*^54^ evaluated ferroptosis-related markers, identifying PNI as the only histopathologic factor consistently associated with OS, supporting the role of PNI as a phenotypic indicator of biologically aggressive ICC. Tajima *et al.*^59^ specifically compared ICC with and without an intraductal papillary carcinoma component (IDPCC). PNI was far more frequent in non-IDPCC tumor and remained a very strong independent predictor of poor OS, suggesting that the absence of an intraductal component permits more invasive growth patterns.^62^ Yoo *et al.*^73^ incorporated PNI into analyses of perioperative carbohydrate antigen 19-9 (CA19-9) dynamics, demonstrating that PNI independently predicted OS along with CA19-9 non-normalization, margin status, and nodal metastasis. In an HBV-associated ICC cohort, Ahn *et al.*^19^ focused on the prognostic interactions between viral status and pathological features. They demonstrated that PNI, together with tumor size and LNM, was independently associated with poorer OS and recurrence-free survival (RFS) despite HBV status itself not being prognostic.

Other studies examined PNI in relation to key pathological tumor features, including invasion patterns, tumor morphology, stage-specific risk, and response to adjuvant therapy. Fisher *et al.*^32^ assessed the combined prognostic significance of LVI and PNI to refine selection for adjuvant therapy; both factors independently predicted shorter OS, and the presence of either conferred outcomes similar to node-positive disease. Yamamoto *et al.*^69^ analyzed tumor with mass-forming growth and radiological vascularity, reporting that PNI was independently associated with worse OS along with LNM. In a large multicenter study, Wei *et al.*^67^ comprehensively examined PNI across early-stage and node-negative subgroups and its associations with other pathological findings. PNI-positive tumors exhibited more adverse features, including higher CA19-9 levels, VI and R1 rates, and had significantly shorter OS and disease-free survival. Notably, PNI remained independently prognostic even in American Joint Committee on Cancer (AJCC) T1–2 and N0 subgroups, and was associated with a higher rate of extrahepatic recurrence, highlighting its utility for risk stratification even in ostensibly low-risk disease. Zhang *et al.*^16^ contributed key evidence by specifically analyzing PNI in MF ICC after R0 resection and evaluating whether it modifies the benefit of adjuvant chemotherapy. PNI was common (43%) and independently predicted significantly shorter RFS and OS. Importantly, PNI status moderated chemotherapy benefit: patients without PNI derived clear improvement in both RFS and OS from adjuvant chemotherapy, whereas patients with PNI did not. Thus, PNI may help identify subgroups with differential responsiveness to postoperative therapy. Zou *et al.*^79^ focused exclusively on early-stage ICC (AJCC T1–T2 N0 M0) and demonstrated that PNI is a powerful independent predictor of both RFS and OS in this low-stage population. Their combined T-stage/PNI stratification showed that T1 tumors with PNI behaved clinically like T2 tumors without PNI, whereas T2 tumors with PNI represented the highest-risk group. These results indicate that PNI meaningfully upstages biological risk and may warrant incorporation into early-stage TNM classification.

Two recent studies integrated PNI into broader modelling approaches. Li Q *et al.*^42^ used a machine-learning framework to identify risk factors for early recurrence and long-term survival, revealing PNI to be an independent predictor of early recurrence and a significant factor in both OS and RFS. Li R *et al.*^43^ incorporated PNI into a sarcopenia-adjusted prognostic nomogram and showed that it remained independently associated with OS alongside sarcopenia indices, tumor differentiation, and TNM stage.

## Discussion

In this systematic review and meta-analysis of 61 studies including 15,502 patients, we provide the most comprehensive evaluation to date of PNI in ICC. The pooled prevalence of PNI was approximately 36%, with substantial between-study heterogeneity. Sensitivity analyses confirmed the robustness of this estimate, and meta-regression identified publication year and study size as independent moderators of reported prevalence with newer and larger studies reporting a lower prevalence of PNI. At the study level, PNI clustered with other markers of aggressive tumor biology, showing significant positive correlations with both LNM and VI, and inverse correlation with the proportion of MF subtype. One-third of studies reported a significant association of PNI with OS, and the pooled HR further supported this finding (HR 1.79, 95% CI 1.38–2.19, *p* <0.001).

PNI in ICC is closely associated with the underlying tumor biology. Previous studies have shown that some ICC subtypes are associated with higher rates of PNI than others. Bagante *et al.*^22^ demonstrated that in a large cohort comprising 1,206 patients, PNI was more prevalent in the PI subtype of ICC than in the MF subtype. This finding was subsequently confirmed by studies involving both smaller and similarly sized cohorts.^52^^,^^66^ Our data further support these observations, demonstrating an inverse correlation between PNI and the proportion of the MF subtype. This finding indirectly indicates that higher proportions of PI and IG subtypes are associated with higher rates of PNI. These findings are biologically plausible, as PI tumors tend to be more invasive and less well differentiated; therefore, they are more frequently resected with R1 margins. Given that PNI status often correlates with microscopically positive resection margins (R1), this association may also contribute to the observed differences in PNI prevalence between ICC subtypes. Sigel *et al.*^57^ compared small- and large-duct patterns among ICCs and found that the large-duct pattern was associated with a significantly higher PNI prevalence of 71%. Yamashita *et al.*^70^ highlighted that tumor location had a significant impact on the PNI prevalence, with perihilar tumors exhibiting PNI more frequently than peripheral tumors. Similarly, Ebata *et al.*^30^ corroborated this observation; in their comparative analysis of perihilar ECC and ICC, they reported comparable biological behavior, with a remarkably high PNI prevalence of 90.4% among ICC cases. Conversely, Ke *et al.*^38^ reported a lower proportion of PNI-positive patients (<10%) in their cohort of patients with ICC with clinically negative LNM. These findings support the hypothesis that PNI reflects an overall aggressive tumor phenotype associated with LNM and VI, rather than representing an independent prognostic factor (see section below).

In ECC and pancreatic cancer, PNI is a well-established adverse prognostic factor.^14^^,^^15^ In ICC, however, evidence has been inconsistent. Approximately one-third of studies identified PNI as an independent adverse prognostic factor for OS. Although our meta-analysis supports its overall detrimental impact, the independent impact of PNI on OS remains debatable. Several explanations may account for this inconsistency. First, PNI frequently coexists with other indicators of aggressive tumor biology, such as LNM, VI, and tumor subtypes. The observed prognostic impact of PNI may thus be difficult to disentangle from the underlying stage of disease and, importantly, from intrinsic tumor growth patterns. Jansen *et al.*^37^ demonstrated a significant association of PNI with LNM in ICC—a well-established determinant of poor survival.^80^ Similarly, in gastric cancer, Duraker *et al.*^81^ reported that the presence of PNI correlated with tumor volume, depth of invasion, angiogenesis, and lymph node involvement, supporting the concept that PNI reflects an overall aggressive tumor phenotype rather than a stand-alone prognostic factor. Moreover, in contrast to the well-established impact of PNI in ECC, the absence of a consistent prognostic effect in ICC may reflect underlying biological differences between intrahepatic and extrahepatic bile duct tumors.^82^ The relationship between tumor growth pattern and histopathological subtype, and the potential impact and prevalence of PNI on prognosis in ICC, is a subject of ongoing research. In particular, differences in tumor morphology and histopathological classification, including MF *vs.* PI phenotypes and WHO-based small-duct *vs.* large-duct subtypes, are likely key drivers of both PNI prevalence and clinical outcomes. Large-duct type tumors and PI growth patterns are associated with higher rates of PNI and more aggressive behavior.^34^^,^^57^ Accordingly, PNI may represent a surrogate of these adverse biological features rather than an independent determinant of prognosis.

The substantial variability in reported PNI prevalence across studies appears to be multifactorial, reflecting not only biological differences but also methodological inconsistencies in study design and pathological assessment. Although study size and year of publication were identified as relevant moderators, a substantial proportion of the variability in reported PNI prevalence remained unexplained. This is possibly a result of unmeasured differences across studies, particularly the evolution and inconsistency of pathological sampling strategies and diagnostic definitions over time. Most studies did not specify how PNI status was assessed, suggesting that no standardized sampling strategy was applied. In routine practice, many pathology departments examine only a limited number of tumor blocks (*e.g.* two to three regions) rather than performing extensive sampling owing to financial and workload constraints. This approach can lead to imprecise classification of ICC subtypes and may also reduce the sensitivity for detecting PNI. The variability of reported PNI prevalence may be related to the lack of a concise and universally accepted definition of this pathological process. Early definition by Batsakis^83^ in 1985—*tumor cell invasion in, around, and through the nerves*—left room for interpretation and was not precise. However, Liebig *et al.*^13^ in 2009 suggested a more definite criteria for defining PNI—*finding tumor cells within any of the three layers of the nerve sheath or tumor foci outside of the nerve with involvement of ≥33% of the nerve's circumference are sufficient features for calling PNI*. An adaption of this definition might explain the reduction of PNI prevalence in more recent publications. With regard to sample size, small-study effects should also be considered. Meta-regression demonstrated an inverse association between study size and reported PNI prevalence, indicating that smaller cohorts tended to report higher prevalence estimates than larger cohorts, which may have contributed to the observed heterogeneity. Although the pooled estimate appeared robust in fail-safe N analysis, the presence of significant funnel plot asymmetry indicates that small-study effects may have influenced the results. Nevertheless, leave-one-out sensitivity analyses showed that no individual study exerted a disproportionate influence on the overall pooled prevalence estimate.

### Strengths and limitations

The strengths of this study include its large sample size, systematic approach, robust sensitivity analyses, and detailed moderator exploration. Importantly, multivariable meta-regression demonstrated that nearly half of the heterogeneity in prevalence estimates could be explained by study-level characteristics. Limitations must also be acknowledged. The principal limitation of this study is the high degree of heterogeneity observed in the prevalence analysis, which restricts the clinical interpretability of the pooled prevalence estimate. Most included studies were retrospective and thus subject to inherent biases. Most studies did not describe the definition of PNI or specify how PNI assessment and tissue sampling were performed; additionally, reporting of clinicopathological data was not always consistent. HRs for OS were not consistently reported, thereby limiting the number of studies eligible for quantitative synthesis and potentially introducing selection bias. Moreover, variability in multivariable adjustment across studies may affect the comparability of reported effect estimates. The correlation analyses of PNI with other adverse histopathological features were performed at the study level and therefore reflect ecological associations between study-specific prevalence estimates rather than patient-level co-occurrence of these features. Furthermore, our analysis was based predominantly on studies conducted in Asian populations. Although geographic region was not identified as a significant moderator, this predominance of Asian cohorts may still limit the generalizability of the findings to other populations.

### Conclusions

PNI is present in approximately one-third of patients with ICC, is associated with poorer clinical outcomes, and clusters with other adverse histopathological features, particularly LNM and VI, underscoring its association with aggressive tumor biology. Although these findings are tempered by substantial heterogeneity, they consistently highlight the adverse impact of PNI. Standardized pathological definitions, together with prospective studies employing uniform assessment and reporting, are needed to better define the prognostic and clinical significance of PNI in ICC.

## Abbreviations

AJCC, American Joint Committee on Cancer; CA19-9, carbohydrate antigen 19-9; df, degrees of freedom; ECC, extrahepatic cholangiocarcinoma; HR, hazard ratio; ICC, intrahepatic cholangiocarcinoma; IDPCC, intraductal papillary carcinoma component; IG, intraductal growth; LNM, lymph node metastasis; LVI, lymphovascular invasion; MF, mass-forming; OS, overall survival; PI, periductal-infiltrating; PNI, perineural invasion; PRISMA, Preferred Reporting Items for Systematic Reviews and Meta-Analyses; RFS, recurrence-free survival; VI, vascular invasion.

## Authors’ contributions

Conceptualization: JK, DG, UF, WS, TAA. Data curation: CWP, CH, TAA, JK. Formal analysis: DG, JK, UF. Investigation: all authors. Methodology: JK, DG. Supervision: UF, WS, DG. Visualization: CWP, JK, CH. Writing – original draft: JK, TAA, UF, DG. Writing – review and editing: all authors.

## Data availability

The datasets generated and/or analyzed during the current study are not publicly available because of restrictions related to data ownership, but they are available from the corresponding author on reasonable request.

## Declaration of generative AI and AI-assisted technologies in the manuscript preparation process

During the preparation of this work, the authors used ChatGPT (GPT-5.3 series and GPT-5.4 series) for language editing and internal revision. Following the use of these tools, the authors critically reviewed, revised, and validated the content as necessary and assume full responsibility for the final content and conclusions presented in the published article.

## Financial support

Open Access funding was enabled and organized by Projekt DEAL. No funding was received.

## Conflicts of interest

All authors report that they have no conflict of interest.

Please refer to the accompanying ICMJE disclosure forms for further details.
