# Supplementary material for: Perineural invasion in intrahepatic cholangiocarcinoma: Meta-analysis of prevalence, association with tumor features, and impact on survival
Source: JHEP Rep. 2026 Jun 25;8(9):101939. doi: 10.1016/j.jhepr.2026.101939 (PMC13524604; doi:10.1016/j.jhepr.2026.101939)
Supplement: Supplementary file 1 [file mmc1.pdf]

Perineural invasion in intrahepatic cholangiocarcinoma: meta-analysis of prevalence, association with tumor features, and impact on survival

Johannes Kolck, Timo Alexander Auer, Camilla Winther Pedersen, Clarissa Hosse, Jana Ihlow, Felix Krenzien, Wenzel Schöning, Uli Fehrenbach, Dominik Geisel

Table of content

Table S1……………………………………………………………………………………2

Table S1

Supplementary Table 1: Risk of bias assessment using JBI Critical Appraisal Checklist ratings for all included cohort studies. The 11-item JBI tool evaluates methodological quality across the following domains: (1) similarity of groups and recruitment from the same population; (2) similarity and consistency of exposure measurement used to classify groups; (3) validity and reliability of exposure measurement; (4) identification of important confounding factors; (5) use of appropriate strategies to deal with confounding; (6) confirmation that participants were free of the outcome at the start of follow-up; (7) validity and reliability of outcome measurement; (8) sufficiency and reporting of follow-up time; (9) completeness of follow-up and description of losses; (10) strategies used to address incomplete follow-up; and (11) appropriateness of statistical analysis. Responses were marked as ✔ (criterion met), ✘ (criterion not met), "? "(unclear), and — (not applicable). An overall risk-of-bias judgement was derived based on the number of unmet/unclear items, with categories defined as low risk (0–1 missing items), moderate risk (2–3 missing items), and high risk (≥4 missing items).

Study	1	2	3	4	5	6	7	8	9	10	11	Missing
Ahn 2018	✔	✔	✔	✔	✔	✔	✔	✔	✔	—	✔	0
Aishima 2002	✔	✔	✔	✔	✔	✔	✔	✔	?	?	✔	2
Alaimo 2023	✔	✔	✔	✔	✔	✔	✔	✔	?	?	✔	2
Bagante 2018	✔	✔	✔	✔	✔	✔	✔	✔	?	?	✔	2
Bai 2024	✔	✔	✔	✔	✔	✔	✔	✔	✘	—	✔	1
Bartsch 2021	✔	✔	✔	✔	✔	✔	✔	✔	?	?	✔	2
Bednarsch 2021	✔	✔	✔	✔	✔	✔	✔	✔	?	?	✔	2
Chang 2017	✔	✔	✔	✔	✔	✔	✔	✔	✔	—	✔	0
Chen 2025	✔	✔	✔	✔	✔	✔	✔	✔	?	?	✔	2
Chen 2000	✔	✔	✔	✔	✔	✔	✔	✔	?	?	✔	2
de Franchis 2024	✔	✔	✔	?	?	—	✔	—	—	—	✔	2
Ebata 2009	✔	✔	✔	✔	✔	✔	✔	✔	?	✘	✔	2
Ercolani 2015	✔	✔	✔	✔	✔	✔	✔	✔	?	✘	✔	2
Fisher 2012	✔	✔	✔	✔	✔	✔	✔	✔	?	✘	✔	2
Fujita 2017	✔	✔	✔	✔	✔	✔	✔	✔	✘	✘	✔	2
Hayashi 2016	✔	✔	✔	✔	✔	✔	✔	✔	✘	✘	✔	2
Hirohashi 2002	✔	✔	✔	✔	✘	✔	✔	✔	?	✘	✔	3
Hu 2019	✔	✔	✔	✔	✔	✔	✔	✔	?	✘	✔	2
Jansen 2018	✔	✔	✔	✔	✘	✔	✔	✔	?	✘	✔	3
Ke 2021	✔	✔	✔	✔	✔	✔	✔	✔	✘	✘	✔	2
Kubo 2014	✔	✔	✔	✔	✘	✔	✔	✔	?	✘	✔	3
Kuroda 2007	✔	✔	✔	✔	✔	✔	✔	✔	?	✘	✔	2
Lee 2014	✔	✔	✔	✔	✔	✔	✔	✔	✘	✘	✔	2
Li 2025	✔	✔	✔	✔	✔	✔	✔	✔	✔	—	✔	0
Li 2024	✔	✔	✔	✔	✔	✔	✔	✔	?	✘	✔	2
Liang 2017	✔	✔	✔	✔	✔	✔	✔	✔	?	✘	✔	2
Liao 2025	✔	✔	✔	✔	✔	✔	✔	✔	?	✘	✔	2
Liu 2024	✔	✔	✔	✔	✔	✔	✔	✔	?	✘	✔	2
Ma 2024	✔	✔	✔	✔	✔	✔	✔	✔	✔	✘	✔	1
Mano 2013	✔	✔	✔	✔	✔	✔	✔	✔	?	✘	✔	2
Misumi 2016	✔	✔	✔	✔	✔	✔	✔	✔	?	✘	✔	2
Qi 2025	✔	✔	✔	✔	✔	✔	✔	—	✔	—	✔	0
Raghavan 2015	✔	✔	✔	✘	✘	✔	✔	—	✔	—	✔	2
Rhee 2019	✔	✔	✔	✘	✘	✔	✔	✔	✔	✘	✔	3
Sakata 2019	✔	✔	✔	✔	✔	✔	✔	✔	?	✘	✔	2
Sarcognato 2022	✔	✔	✔	✔	✔	✔	✔	✔	?	✘	✔	2
Shinozaki 2011	✔	✔	✔	✔	✔	✔	✔	✔	?	✘	✔	2
Shirai 2008	✔	✔	✔	✔	✔	✔	✔	✔	✔	✘	✔	1
