# Supplementary material for: Perineural invasion in intrahepatic cholangiocarcinoma: Meta-analysis of prevalence, association with tumor features, and impact on survival
Source: JHEP Rep. 2026 Jun 25;8(9):101939. doi: 10.1016/j.jhepr.2026.101939 (PMC13524604; doi:10.1016/j.jhepr.2026.101939)
Supplement: Supplementary file 2 [file mmc2.pdf]

JHEP Reports

CTAT methods

Tables for a "Complete, Transparent, Accurate and Timely account" (CTAT) are now mandatory for all revised submissions. The aim is to enhance the reproducibility of methods.

- Only include the parts relevant to your study
- Refer to the CTAT in the main text as 'Supplementary CTAT Table'
- Do not add subheadings
- Add as many rows as needed to include all information
- Only include one item per row

If the CTAT form is not relevant to your study, please outline the reasons why:

This CTAT is partially applicable. This manuscript is a systematic review and meta-analysis using published literature and software-based analyses. No antibodies, cell lines, organisms, sequence-based reagents, biological samples, drugs, proteins, or vectors were used.

1.1 Antibodies

Name	Citation	Supplier	Cat no.	Clone no.
Not applicable	—	—	—	—

1.2 Cell lines

Name	Citation	Supplier	Cat no.	Passage no.	Authentication test method
Not applicable	—	—	—	—	—

1.3 Organisms

Name	Citation	Supplier	Strain	Sex	Age	Overall n number
Not applicable	—	—	—	—	—	—

1.4 Sequence based reagents

Name	Sequence	Supplier
Not applicable	—	—

1.5 Biological samples

Description	Source	Identifier
Published study-level data from eligible ICC cohorts	Included full-text articles from PubMed, Web of Science, and Cochrane Library	Data extraction for meta-analysis; no new samples collected

1.6 Deposited data

Name of repository	Identifier	Link
OSF Registries (protocol registration)	DOI 10.17605/OSF.IO/YN4UB	https://osf.io/yn4ub
No public data repository for extracted dataset	Not publicly available	Data available from corresponding author on reasonable request

1.7 Software

Software name	Manufacturer	Version
R	R Foundation for Statistical Computing	Version not reported in manuscript
PubMed	National Library of Medicine / NCBI	Searched through 31 Aug 2025
Web of Science	Clarivate	Searched through 31 Aug 2025
Cochrane Library	Cochrane	Searched through 31 Aug 2025

1.8 Other (*e.g.* drugs, proteins, vectors etc.)

PRISMA statement	Reference [17]	Reporting guideline
JBI critical appraisal tool for cohort studies	Reference [18]	Risk-of-bias tool
Random-effects meta-analysis	Methods described in manuscript	Primary pooling approach
OSF Registries	DOI 10.17605/OSF.IO/YN4UB	Preregistered protocol

1.9 Please provide the details of the corresponding methods author for the manuscript:

Dr. med. Johannes Kolck, MD
Charité - Universitätsmedizin Berlin, Department of Radiology
Augustenburger Platz 1, 13353 Berlin, Germany
Tel.: +49450360147
Email: Johannes.kolck@charite.de

2.0 Please confirm for randomised controlled trials all versions of the clinical protocol are included in the submission. These will be published online as supplementary information.

Not applicable. This submission is a systematic review and meta-analysis, not a randomised controlled trial.
