# Supplementary material for: Perineural invasion in intrahepatic cholangiocarcinoma: Meta-analysis of prevalence, association with tumor features, and impact on survival
Source: JHEP Rep. 2026 Jun 25;8(9):101939. doi: 10.1016/j.jhepr.2026.101939 (PMC13524604; doi:10.1016/j.jhepr.2026.101939)
Supplement: Supplementary file 4 [file mmc4.pdf]

Research article

JHEP Reports

Perineural invasion in intrahepatic cholangiocarcinoma: Meta-analysis of prevalence, association with tumor features, and impact on survival

Authors

Johannes Kolck, Timo Alexander Auer, Camilla Winther Pedersen, …, Wenzel Schöning, Uli Fehrenbach, Dominik Geisel

Correspondence

Johannes.kolck@charite.de (J. Kolck).

Graphical abstract

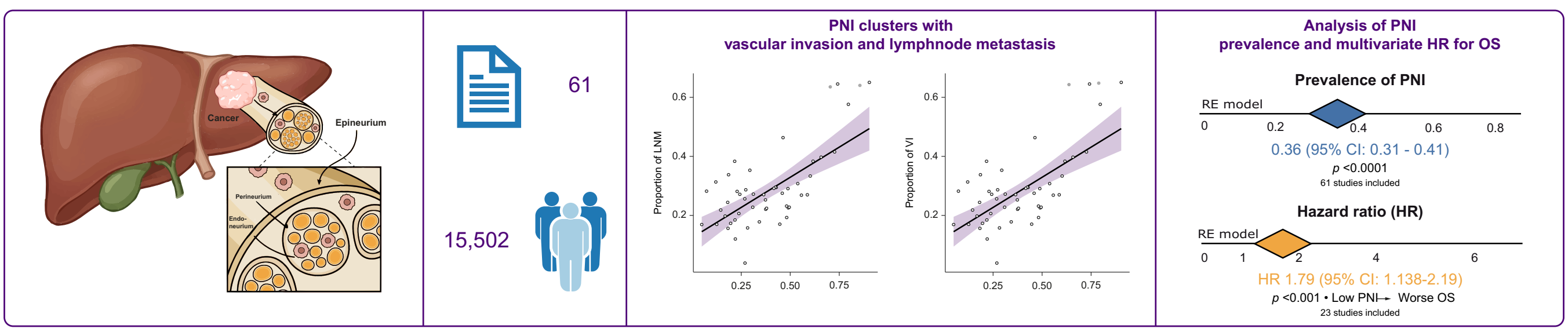

PNI in ICC is common, co-occurs with lymph node metastasis, vascular invasion, and adverse tumor subtypes, and is associated with poor outcomes. It may represent a surrogate marker of aggressive tumor biology rather than an independent driver of prognosis

Highlights:

- Perineural invasion (PNI) occurs in approximately one-third of patients with resected intrahepatic cholangiocarcinoma.
- PNI clusters with adverse tumor features, such as lymph node metastasis and vascular invasion, and is associated with adverse survival outcomes.
- These findings suggest that PNI may reflect aggressive tumor biology rather than act as an independent prognostic factor.

Impact and implications:

Perineural invasion (PNI) is a proposed marker of tumor aggressiveness in intrahepatic cholangiocarcinoma (ICC); however, its prevalence and prognostic significance have remained uncertain, warranting systematic evaluation. This study provides the most comprehensive synthesis to date of PNI in ICC demonstrating that PNI occurs in approximately one-third of ICC cases and clusters with adverse pathological features, although its independent impact on survival remains debatable. These findings are relevant for clinicians and researchers involved in ICC management, as they suggest that PNI may contribute to more refined risk stratification, particularly in early-stage disease. In practice, PNI assessment could support clinical decision-making and patient counseling. However, future standardized and prospective studies are needed to clarify its prognostic value and potential role in guiding treatment strategies.

https://doi.org/10.1016/j.jhepr.2026.101939

© 2026 The Author(s). Published by Elsevier B.V. on behalf of European Association for the Study of the Liver (EASL). This is an open access article under the CC BY license (http://creativecommons.org/licenses/by/4.0/). JHEP Reports, 2026, 8, 1–10

JHEP|Reports

Perineural invasion in intrahepatic cholangiocarcinoma: Meta-analysis of prevalence, association with tumor features, and impact on survival

Johannes Kolck[1,*], Timo Alexander Auer[1], Camilla Winther Pedersen[2], Clarissa Hosse[1], Jana Ihlow[3], Felix Krenzien[4], Wenzel Schöning[4], Uli Fehrenbach[1,†], Dominik Geisel[1,†]

JHEP Reports **2026**. vol. 8 | 1–10

Check for updates

Background & Aims: Perineural invasion (PNI) is a recognized marker of tumor aggressiveness in several malignancies, but its prevalence and prognostic significance in intrahepatic cholangiocarcinoma (ICC) remain uncertain. This systematic review and meta-analysis aimed to estimate the pooled prevalence of PNI and evaluate its association with overall survival (OS) in ICC.

Methods: Following PRISMA guidelines, PubMed, Web of Science, and the Cochrane Library were searched through August 2025. The PNI prevalence was pooled using a random-effects model. Where available, multivariable hazard ratios for OS were pooled using a random-effects meta-analysis. Heterogeneity was assessed and potential moderators were explored using meta-regression. Pairwise ecological correlations between PNI and other tumor features were analyzed using Pearson's correlation coefficient.

Results: We analyzed 61 studies including 15,502 patients. The pooled prevalence of PNI was 36.0% (95% CI 31.0–41.0%), with substantial heterogeneity (I^2 = 98.5%). Meta-regression identified publication year and study size as independent moderators, with more recent and larger studies reporting lower PNI prevalence. PNI positively correlated with lymph node metastasis, lymphovascular invasion, and vascular invasion (all p <0.001), and inversely correlated with the proportion of mass-forming ICC (p = 0.025). Of 44 studies assessing OS, 23 were included in meta-analysis, demonstrating that PNI was associated with worse survival (hazard ratio 1.79, 95% CI 1.38–2.19, p <0.001).

Conclusions: PNI occurs in approximately one-third of patients with ICC, is associated with poorer outcomes, and clusters with lymph node metastasis and vascular invasion, reflecting aggressive tumor biology. Despite the heterogeneity of the available data, its adverse prognostic impact is consistent.

© 2026 The Author(s). Published by Elsevier B.V. on behalf of European Association for the Study of the Liver (EASL). This is an open access article under the CC BY license (http://creativecommons.org/licenses/by/4.0/).

Introduction

Intrahepatic cholangiocarcinoma (ICC) is the second most common primary hepatic malignancy after hepatocellular carcinoma, and its incidence has been steadily increasing worldwide over the past decades.[1] ICC typically arises from second-order bile ducts, shows aggressive behavior, and often remains asymptomatic until advanced stages.[2,3] Three main types of ICC are recognized: (1) mass-forming (MF), which appears as a distinct mass in the liver parenchyma; (2) periductal-infiltrating (PI), which spreads longitudinally along the bile duct, often causing upstream dilatation; and (3) intraductal growth (IG), which grows papillarily into the duct lumen, sometimes resembling a tumor thrombus.[4] At diagnosis, up to 54% of patients have unresectable tumors, whereas only ~35% qualify for surgery, the mainstay of curative intended therapies.[5–7] Because of the lack of effective treatment and the high rate of recurrence (57.9–73.4%), the prognosis after resection in these patients is dismal, with a 5-yr survival rate of only 20–40%.[8,9]

Prognostic stratification in ICC is therefore of critical clinical importance. Beyond established factors such as tumor size, vascular invasion (VI), and lymph node metastasis (LNM), perineural invasion (PNI) has increasingly been recognized as a potential histopathological marker of tumor aggressiveness.[10–12]

PNI is defined as the invasion of tumor cells into the perineural space, representing a distinct route of tumor dissemination (Fig. 1) compared with vascular or lymphatic spread.[13]

In extrahepatic cholangiocarcinoma (ECC), the presence of PNI has consistently been associated with poor prognosis.[14,15] In ICC, however, the prognostic impact of PNI is less clear, with studies reporting widely variable prevalence rates and inconsistent associations with overall survival (OS).[16]

Given these discrepancies, a systematic synthesis of the available evidence is warranted. Therefore, we conducted the

* Corresponding author. Address: Charité – Universitätsmedizin Berlin, corporate member of Freie Universität Berlin and Humboldt-Universität zu Berlin, Department of Radiology, Augustenburger Platz 1, 13353 Berlin, Germany. Tel.: +49450360147.
E-mail address: Johannes.kolck@charite.de (J. Kolck).
† These authors contributed equally to this work.
https://doi.org/10.1016/j.jhepr.2026.101939

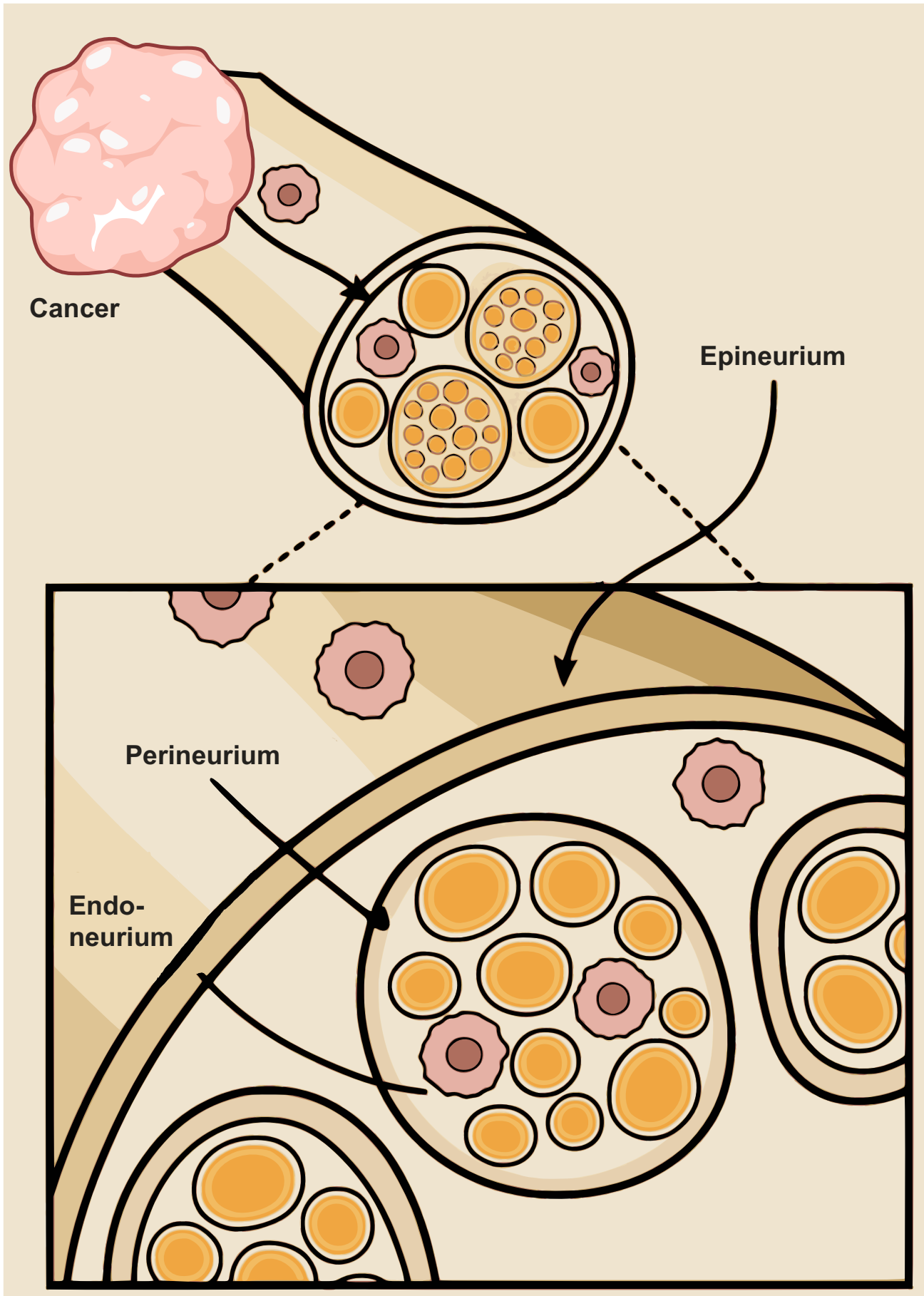

Fig. 1. Proportional forest plot illustrating the prevalence of perineural invasion in intrahepatic cholangiocarcinoma. Across 61 studies, the pooled prevalence was 36.0% (95% CI 31.0–41.0%) using a random-effects model. Substantial between-study heterogeneity was observed (Cochran's Q = 2943.41, df = 60, p <0.01; I^2 = 98.47%; τ^2 = 0.182), supporting the use of the random-effects model. PNI, perineural invasion. Adapted from Bahmad *et al.*[[84]]

present meta-analysis with two main aims: (1) to define the pooled prevalence of PNI in ICC, and (2) to investigate the association between PNI and OS in patients with ICC.

Materials and methods

Search strategy and study selection

We conducted this systematic review and meta-analysis in accordance with the Preferred Reporting Items for Systematic Reviews and Meta-Analyses (PRISMA) guidelines.[17] The protocol was preregistered on Open Science Framework Registries before data extraction/analysis (DOI 10.17605/OSF.IO/YN4UB, 05 September 2025). A comprehensive literature search was performed in PubMed, Web of Science, and the Cochrane Library up to 31 August 2025. The search strategy combined controlled vocabulary terms, where available (*e.g.* MeSH terms in PubMed), with free-text keywords and their combinations, including 'intrahepatic cholangiocarcinoma', 'intrahepatic bile duct cancer', 'ICC', 'perineural invasion', and 'PNI'. Only articles in English language were selected.

Studies were included if they met the following criteria: (1) patients were diagnosed with ICC (histologically confirmed); (2) PNI was assessed histopathologically; and (3) the prevalence of PNI was reported.

Exclusion criteria comprised case reports, reviews, conference abstracts, studies with fewer than 20 patients, and those not differentiating ICC from other cholangiocarcinoma subtypes.

Data extraction and quality assessment

Two reviewers (CWP and JK) independently screened titles and abstracts, assessed full texts, and extracted data, with discrepancies resolved by a third reviewer (CH). Extracted variables included study characteristics (first author, year, country, design, and cohort size), patient and tumor features (age, sex, tumor size, PNI status, LNM, lymphovascular [LVI], and VI), and survival outcomes. Study quality was assessed using the JBI critical appraisal tool for the assessment of risk of bias for cohort studies.[18] To assess the impact of PNI on OS, studies were categorized into three groups: (1) PNI not included in OS analysis or OS not analyzed; (2) OS analyzed but PNI not significant; and (3) OS analyzed and PNI significant.

Statistical analysis

The pooled prevalence of PNI across studies was estimated using both fixed- and random-effects models. Owing to substantial between-study variability, the random-effects model was selected as the primary approach. Proportions were logit-transformed before pooling. Between-study heterogeneity was assessed using Cochran's Q statistic, the I^2 statistic, and τ^2. An I^2 value >50% was considered indicative of substantial heterogeneity. To assess robustness, leave-one-out sensitivity analyses were performed in which each study was sequentially excluded and the pooled prevalence recalculated.

To evaluate the role of PNI as a prognostic factor for OS, multivariable hazard ratios (HRs) were extracted from the original articles, and a random-effects meta-analysis was performed. When not directly reported, SEs were derived from the corresponding 95% CIs using established methods described in the Cochrane Handbook for Systematic Reviews of Interventions. Moderator analyses were performed using univariable and multivariable random-effects meta-regression models.

The association of PNI with other tumor characteristics was assessed using study-level prevalence of PNI, LNM, VI, poor differentiation (≥G3), and the proportion of MF tumors, which were extracted from each included study. Pairwise ecological correlations between these variables were evaluated using Pearson's correlation coefficient. Analyses were restricted to studies with available data. Studies reporting only a single subtype of ICC were excluded from the correlation analysis. All analyses were conducted using R version 4.4.2 (R Foundation for Statistical Computing, Vienna, Austria), with p values <0.05 considered statistically significant.

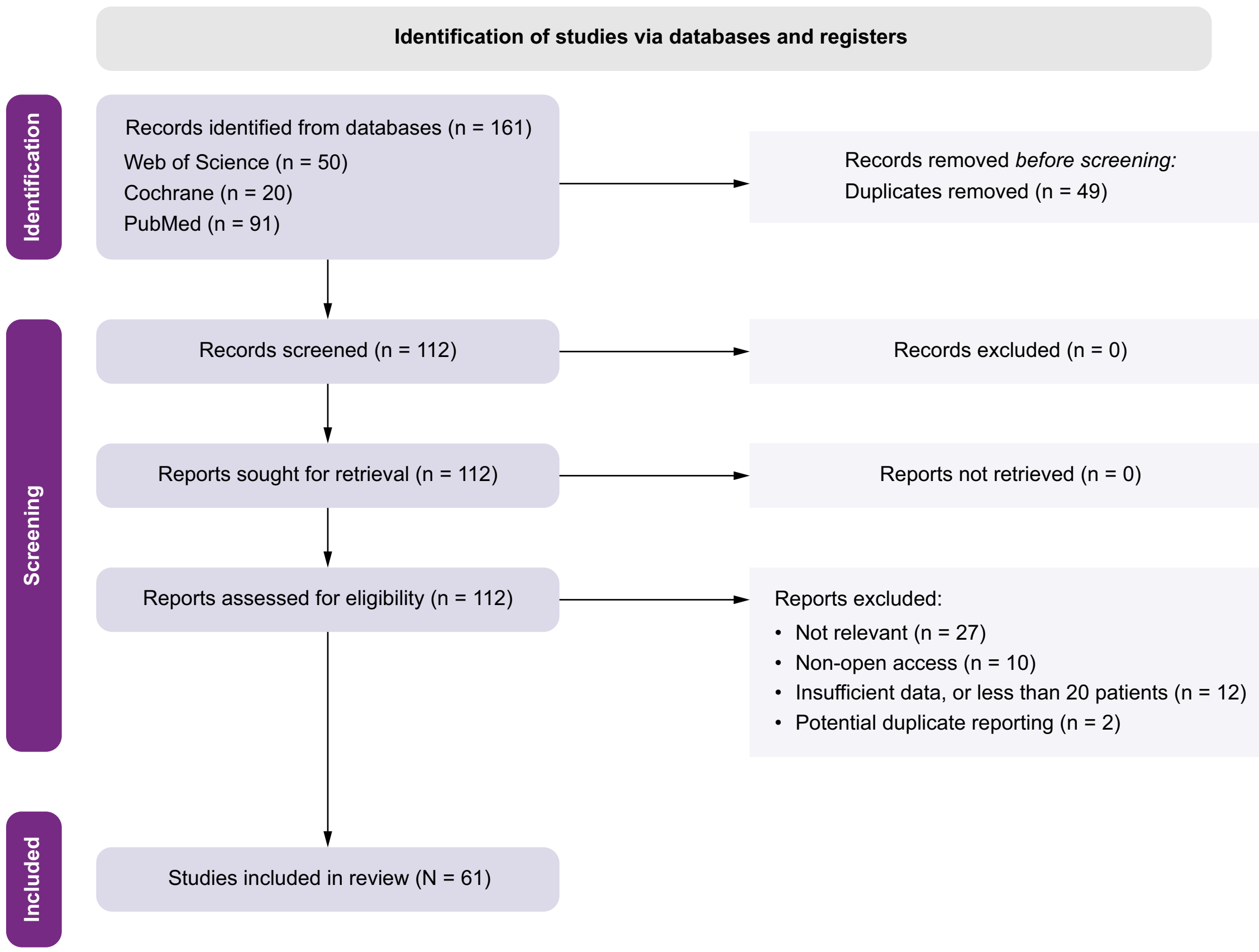

Fig. 2. Flow diagram illustrating the selection process of studies included in the meta-analysis.

Results

Study cohort

A total of 61 retrospective studies with 15,502 patients were included in the meta-analysis.[16,19–79] The study selection process is illustrated by the PRISMA flow chart (Fig. 2). The median cohort size was 103 patients (range 20–1,728). The mean age reported in 48 studies was 62.0 yr (SD 2.9; range 54–68 yr). Sex distribution was available in 57 studies. Overall, 59% of patients were male and 41% were female. The majority of studies was based on patient cohorts in Asia (72.13%). Tumor size and further clinicopathological characteristics (*e.g.* LNM, VI) are reported in Table 1. A minority of studies indicated the application of adjuvant therapy, whereas neoadjuvant treatment was not reported. Risk-of-bias analysis revealed that nearly all included studies fulfilled the critical domains relevant to the primary outcome, indicating that the pooled prevalence estimate was derived predominantly from methodologically valid data (Table S1).

Table 1. Descriptive statistics of included studies.

	Mean	SD	Minimum	Maximum	Missing
No pts	254.13	361.10	20.00	1,728.00	0
PNI (%)	36.4	18.6	4.9	90.4	0
Age (years)	62.01	2.94	54.00	68.00	13
Male (%)	56.7	8.2	34.5	72.3	4
Tumor size (cm)	5.71	1.02	4.00	8.00	23
MF (%)	72.5	16.4	35.5	95.8	38
PD (%)	29.9	18.4	2.6	81.8	9
LNM (%)	27.9	11.8	3.8	65.1	12
VI (%)	41.9	22.8	5.3	97.6	28
LVI (%)	46.3	21.7	13.1	97.1	32
Adjuvant chemotherapy (%)	33.2	10.6	12.7	66.0	42
Publication year	2,017.2	6.1	2,002.0	2,025.0	0

The prevalence of certain features, including adjuvant chemotherapy, was reported in only a limited number of studies and is therefore of limited generalizability. LNM, lymph node metastasis; LVI, lymphovascular invasion; MF, mass-forming ICC subtype; PD, poor differentiation; PNI, perineural invasion, VI, vascular invasion.

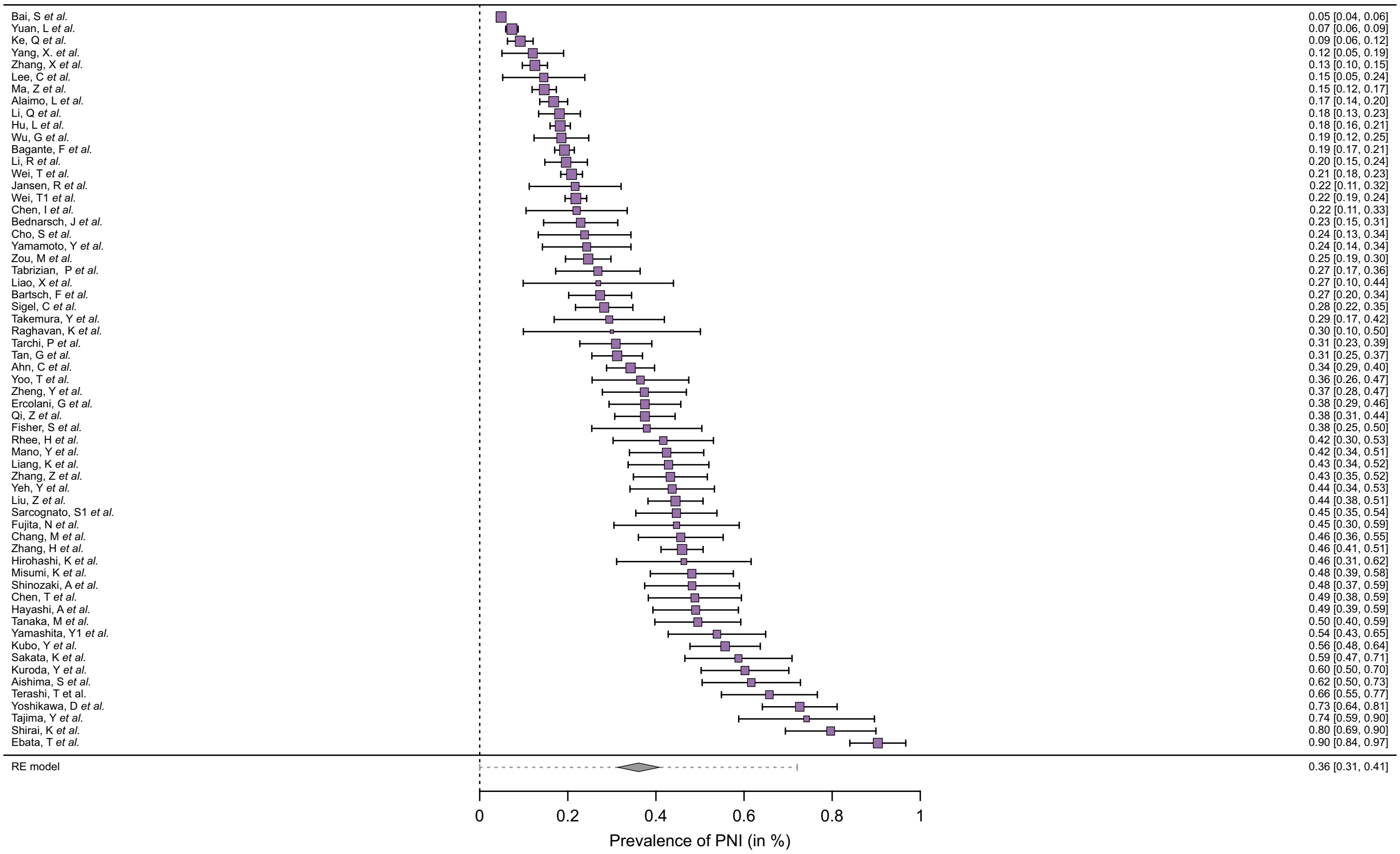

Fig. 3. Proportional forest plot illustrating the prevalence of perineural invasion in intrahepatic cholangiocarcinoma. PNI, perineural invasion.

Prevalence of PNI

Across 61 studies, the pooled prevalence of PNI was 36.0% (95% CI 31.0–41.0%) under a random-effects model. Cochran's Q = 2943.41 (degrees of freedom [df] = 60, p <0.01), I^2 = 98.47%, and τ^2 = 0.182, indicating high between-study heterogeneity and supporting the use of random-effects models (Fig. 3). Sensitivity analysis implementing a leave-one-out approach demonstrated that the pooled prevalence of PNI was highly robust. Excluding each study in turn yielded pooled prevalence estimates ranging from 35.1% to 36.6% compared with the overall estimate of 36.0% (95% CI 31.0–41.0%). This indicates that no single study exerted a disproportionate influence on the summary effect. Assessment of small-study effects showed a large Rosenthal fail-safe N (123,014), suggesting that the pooled result would require many null studies to lose statistical significance. However, both the rank correlation test (Kendall's tau = 0.201, p = 0.022) and Egger's regression test (Z = 4.344, p <0.001) were significant, indicating statistical evidence of funnel plot asymmetry and possible small-study effects. Because funnel plot asymmetry is not specific to publication bias, these findings should be interpreted cautiously.

Moderator analysis (PNI prevalence)

Univariable random-effects meta-regressions (Paule-Mandel τ^2, Knapp-Hartung inference) identified several significant continuous moderators of PNI prevalence. Publication year was strongly associated with lower reported prevalence (β = -0.118, p <0.001), explaining ~35% of between-study heterogeneity. Similarly, larger study size (log-transformed N) predicted lower PNI prevalence (β = -0.092, p <0.001), accounting for ~27% of heterogeneity. Mean cohort age showed a modest positive association (β = +0.099, p = 0.015), but explained only ~11% of heterogeneity. The cohort location (Asia, USA, Europe) was not a significant moderator.

In the multivariable model including publication year, study size, and age simultaneously, only publication year (β = –0.084, p <0.001) and study size (β = -0.057, p = 0.014) remained significant independent predictors of PNI prevalence (Table 2),

Table 2. Results of univariable and multivariable random-effects meta-regression analyses exploring sources of heterogeneity. The multivariable model included all three covariates simultaneously. Publication year and study size remained significant independent predictors of PNI prevalence, whereas age was not independently associated after adjustment. Bold *p* values indicate statistically significant associations (*p* <0.05).

		Univariate				Multivariate			
**Moderator**	**k**	**Coefficient**	**SE**	**z**	***p* value**	**Coefficient**	**SE**	**z**	***p* value**
Publication year	61	-0.118	0.019	-6.121	**<0.001**	-0.084	0.021	-3.995	**<0.001**
Study size	61	-0.092	0.020	-4.487	**<0.001**	-0.058	0.023	-2.465	**0.014**
Region	61	0.217	0.090	2.409	0.076	0.016	0.024	0.658	0.511
Patient age	48	0.054	0.025	2.113	**0.035**	0.051	0.083	0.613	0.540

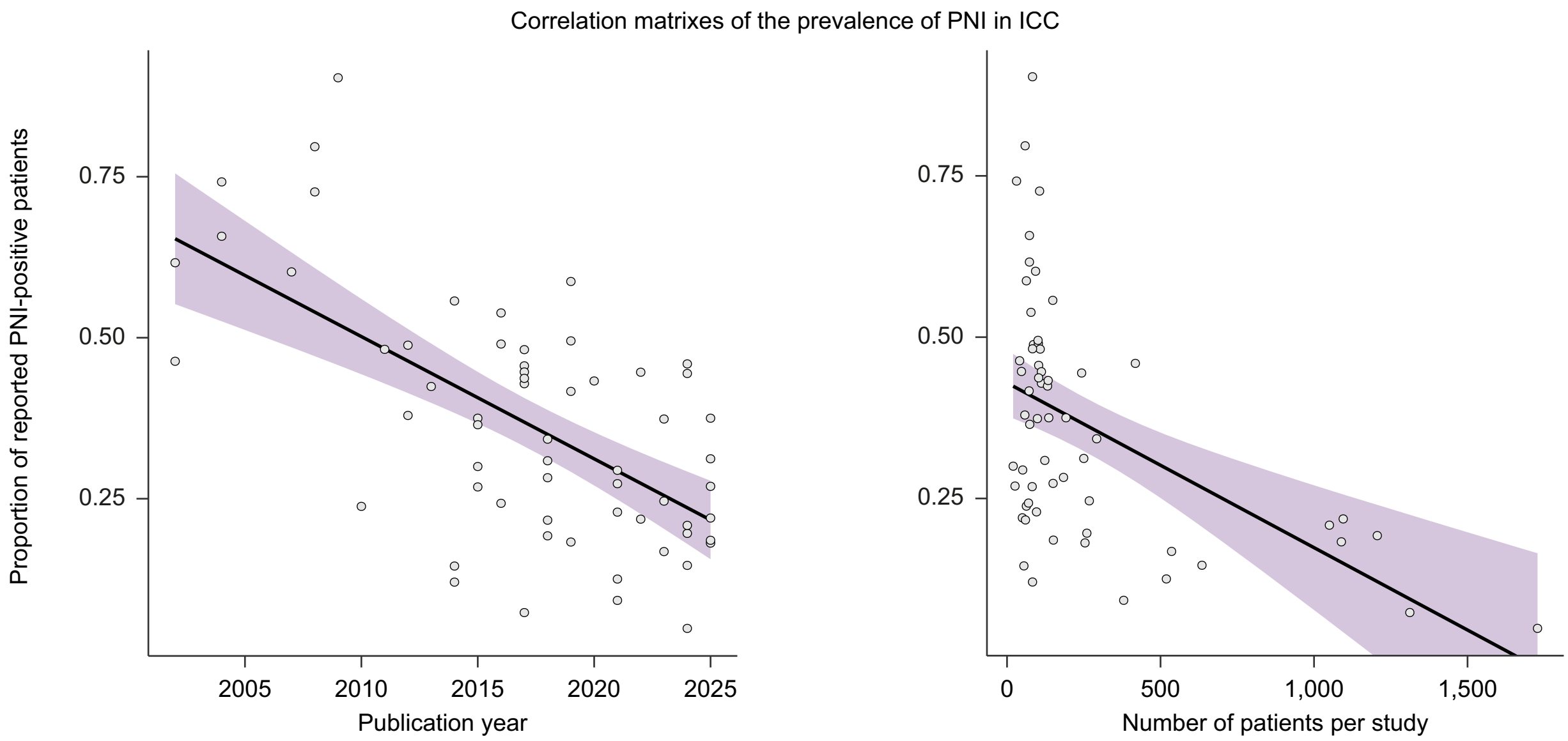

Fig. 4. Correlation matrixes illustrating the two identified sources of heterogeneity (publication year; *p* <0.001) and study size; *p* = 0.014) in the prevalence of PNI in ICC. ICC, intrahepatic cholangiocarcinoma; PNI, perineural invasion.

together explaining ~46% of between-study heterogeneity. Age did not retain significance after adjustment. The correlation of PNI prevalence with publication year and study size is illustrated in Fig. 4.

Correlation of PNI with other features

Study-level correlation analysis demonstrated that PNI prevalence was significantly positively correlated with LNM (r = 0.671, *p* <0.001), VI (r = 0.548, *p* <0.001), and LVI (r = 0.671, *p* <0.001). LNM prevalence was also significantly correlated with LVI (r = 0.482, *p* = 0.013), but not with VI (r = 0.212, *p* = 0.261). The correlation between VI and LVI did not reach statistical significance (r = 0.377, *p* = 0.150). Poor differentiation was not significantly correlated with PNI (r = 0.075, *p* = 0.599), LNM (r = −0.113, *p* = 0.470), VI (r = 0.174, *p* = 0.342), or LVI (r = −0.090, *p* = 0.660).

Conversely, the proportion of the MF subtype was inversely correlated with PNI (r = −0.467, *p* = 0.025) and LNM (r = −0.563, *p* = 0.023), but showed no statistically significant correlation with VI (r = −0.342, *p* = 0.334), LVI (r = −0.380, *p* = 0.200), and poor differentiation (r = 0.187, *p* = 0.405) (Fig. 5 and Table 3).

Impact on overall survival

Out of 44 studies assessing OS, 23 provided sufficient data for inclusion in the meta-analysis; of these 23, 15 studies comprising 4,111 patients reported PNI as significant for OS in multivariate analysis. Using a random-effects model, the presence of PNI was significantly associated with worse OS. The pooled HR was 1.79 (95% CI 1.38–2.19, *p* <0.001), indicating that patients with PNI had a significantly higher hazard of death compared with those without PNI (Fig. 6).

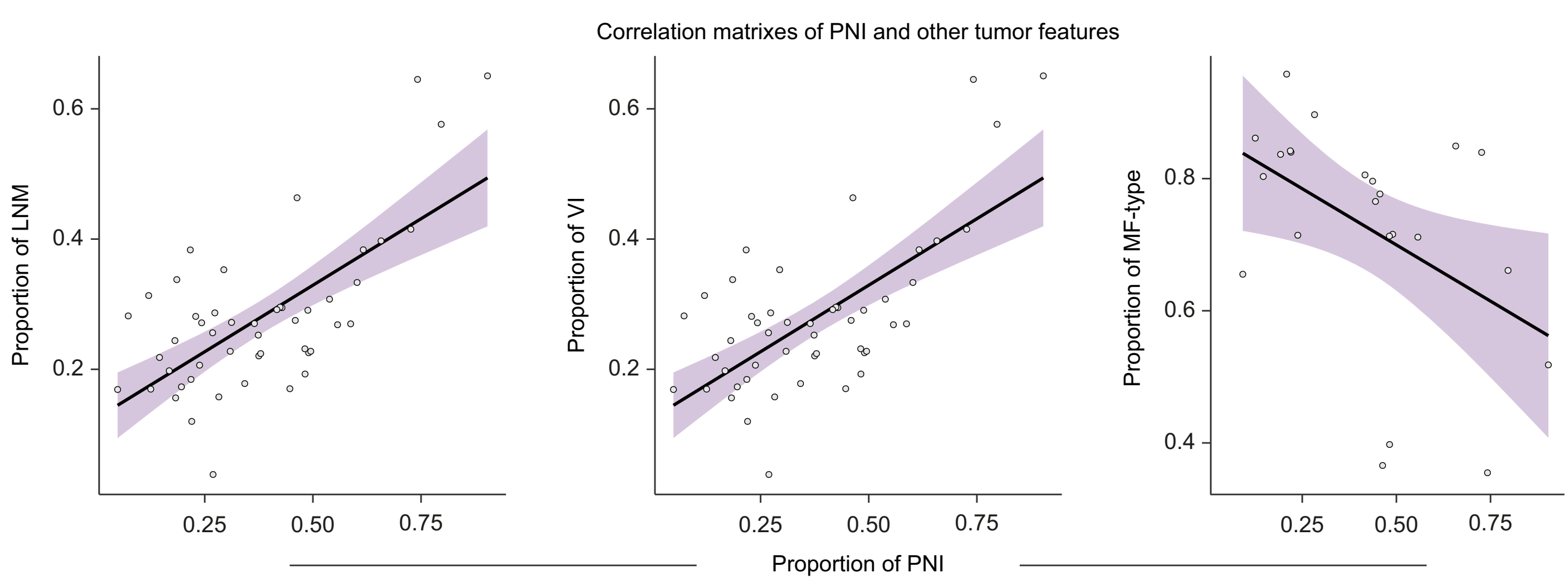

Fig. 5. Study-level correlation analysis demonstrating that PNI prevalence was significantly positively correlated with LNM (*p* <0.001) and VI (*p* <0.001) and inversely correlated with the proportion of MF subtype (*p* = 0.025). LNM, lymph node metastasis; MF, mass-forming; PNI, perineural invasion; VI, vascular invasion.

Table 3. Correlation matrix of PNI with other tumor features.

		PNI (%)	LNM (%)	VI (%)	LVI (%)	PD (%)	MF (%)
PNI (%)	Pearson's r	–					
	df	–					
	p value	–					
LNM (%)	Pearson's r	0.671***	–				
	df	47	–				
	p value	<0.001	–				
VI (%)	Pearson's r	0.548***	0.212	–			
	df	31	28	–			
	p value	<0.001	0.261	–			
LVI (%)	Pearson's r	0.671***	0.482*	0.377	–		
	df	27	24	14	–		
	p value	<0.001	0.013	0.150	–		
PD (%)	Pearson's r	0.075	-0.113	0.174	-0.090	–	
	df	50	41	30	24	–	
	p value	0.599	0.470	0.342	0.660	–	
MF (%)	Pearson's r	-0.467*	-0.563*	-0.342	-0.380	0.187	–
	df	21	14	8	11	20	–
	p value	0.025	0.023	0.334	0.200	0.405	–

Note: *p <0.05, **p <0.01, and ***p <0.001. LNM, lymph node metastasis; LVI, lymphovascular invasion; MF, mass-forming ICC subtype; PD, poor differentiation; PNI, perineural invasion, VI, vascular invasion.

Several studies investigated the molecular and biological correlates of PNI, examining how tumor genetics, phenotypic markers, and growth patterns relate to its prognostic significance. Liang *et al.*[44] examined the GALNT14 genotype and found PNI to be strongly associated with reduced OS independently of molecular subtype, periductal invasion, and mixed histology. Sarcognato *et al.*[54] evaluated ferroptosis-related markers, identifying PNI as the only histopathologic factor consistently associated with OS, supporting the role of PNI as a phenotypic indicator of biologically aggressive ICC. Tajima *et al.*[59] specifically compared ICC with and without an intraductal papillary carcinoma component (IDPCC). PNI was far more frequent in non-IDPCC tumor and remained a very strong independent predictor of poor OS, suggesting that the absence of an intraductal component permits more invasive growth patterns.[62] Yoo *et al.*[73] incorporated PNI into analyses of perioperative carbohydrate antigen 19-9 (CA19-9) dynamics, demonstrating that PNI independently predicted OS along with CA19-9 non-normalization, margin status, and nodal metastasis. In an HBV-associated ICC cohort, Ahn *et al.*[19] focused on the prognostic interactions between viral status and pathological features. They demonstrated that PNI, together with tumor size and LNM, was independently associated with poorer OS and recurrence-free survival (RFS) despite HBV status itself not being prognostic.

Other studies examined PNI in relation to key pathological tumor features, including invasion patterns, tumor morphology, stage-specific risk, and response to adjuvant therapy. Fisher *et al.*[32] assessed the combined prognostic significance of LVI and PNI to refine selection for adjuvant therapy; both factors independently predicted shorter OS, and the presence of either conferred outcomes similar to node-positive disease. Yamamoto

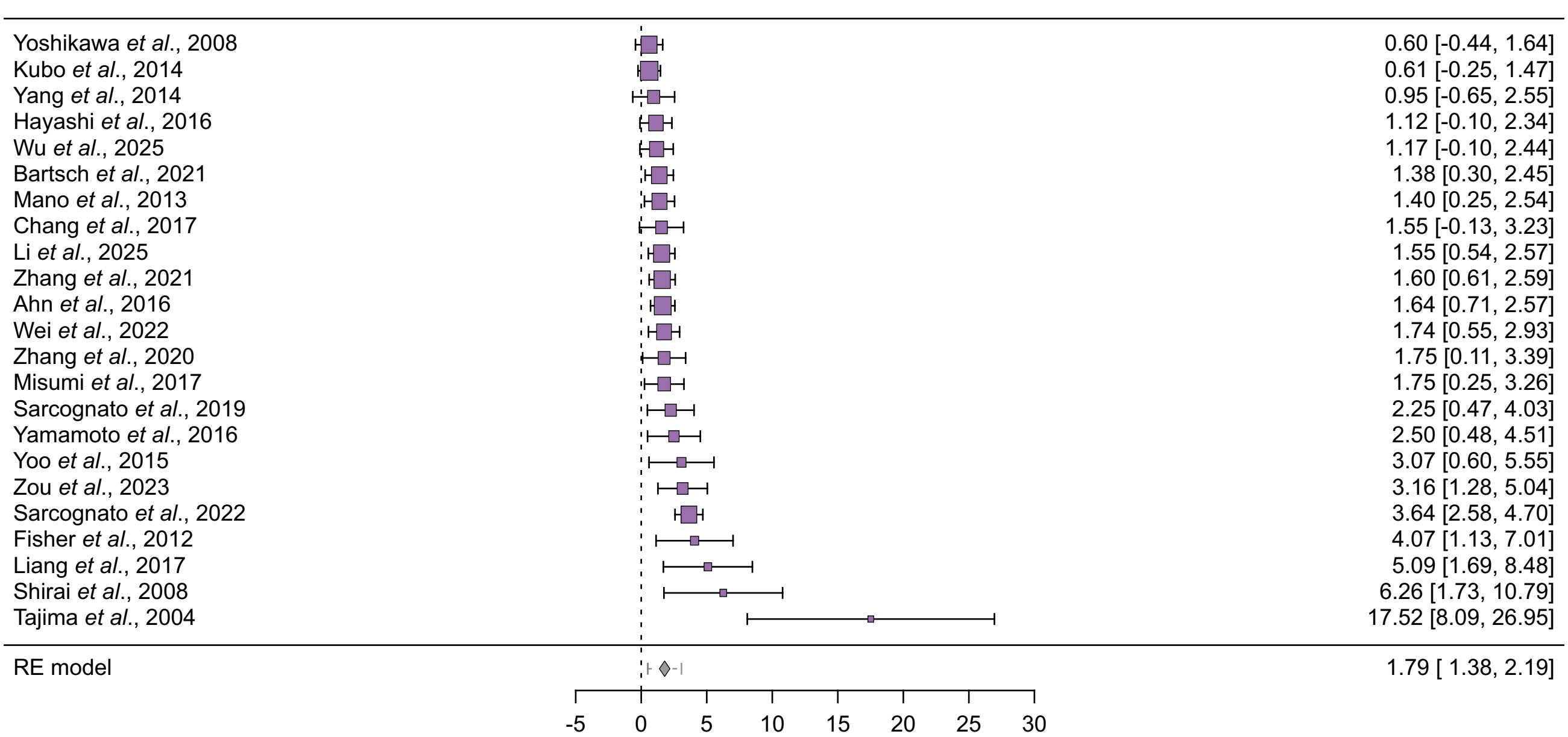

Fig. 6. Forest plot of multivariable hazard ratios for overall survival. A random-effects model including 23 studies demonstrated a significant overall effect (estimate = 1.79, SE = 0.206, Z = 8.67, p <0.001), with a 95% CI ranging from 1.383 to 2.190.

et al.[69] analyzed tumor with mass-forming growth and radiological vascularity, reporting that PNI was independently associated with worse OS along with LNM. In a large multicenter study, Wei et al.[67] comprehensively examined PNI across early-stage and node-negative subgroups and its associations with other pathological findings. PNI-positive tumors exhibited more adverse features, including higher CA19-9 levels, VI and R1 rates, and had significantly shorter OS and disease-free survival. Notably, PNI remained independently prognostic even in American Joint Committee on Cancer (AJCC) T1–2 and N0 subgroups, and was associated with a higher rate of extrahepatic recurrence, highlighting its utility for risk stratification even in ostensibly low-risk disease. Zhang et al.[16] contributed key evidence by specifically analyzing PNI in MF ICC after R0 resection and evaluating whether it modifies the benefit of adjuvant chemotherapy. PNI was common (43%) and independently predicted significantly shorter RFS and OS. Importantly, PNI status moderated chemotherapy benefit: patients without PNI derived clear improvement in both RFS and OS from adjuvant chemotherapy, whereas patients with PNI did not. Thus, PNI may help identify subgroups with differential responsiveness to postoperative therapy. Zou et al.[79] focused exclusively on early-stage ICC (AJCC T1–T2 N0 M0) and demonstrated that PNI is a powerful independent predictor of both RFS and OS in this low-stage population. Their combined T-stage/PNI stratification showed that T1 tumors with PNI behaved clinically like T2 tumors without PNI, whereas T2 tumors with PNI represented the highest-risk group. These results indicate that PNI meaningfully upstages biological risk and may warrant incorporation into early-stage TNM classification.

Two recent studies integrated PNI into broader modelling approaches. Li Q et al.[42] used a machine-learning framework to identify risk factors for early recurrence and long-term survival, revealing PNI to be an independent predictor of early recurrence and a significant factor in both OS and RFS. Li R et al.[43] incorporated PNI into a sarcopenia-adjusted prognostic nomogram and showed that it remained independently associated with OS alongside sarcopenia indices, tumor differentiation, and TNM stage.

Discussion

In this systematic review and meta-analysis of 61 studies including 15,502 patients, we provide the most comprehensive evaluation to date of PNI in ICC. The pooled prevalence of PNI was approximately 36%, with substantial between-study heterogeneity. Sensitivity analyses confirmed the robustness of this estimate, and meta-regression identified publication year and study size as independent moderators of reported prevalence with newer and larger studies reporting a lower prevalence of PNI. At the study level, PNI clustered with other markers of aggressive tumor biology, showing significant positive correlations with both LNM and VI, and inverse correlation with the proportion of MF subtype. One-third of studies reported a significant association of PNI with OS, and the pooled HR further supported this finding (HR 1.79, 95% CI 1.38–2.19, p <0.001).

PNI in ICC is closely associated with the underlying tumor biology. Previous studies have shown that some ICC subtypes are associated with higher rates of PNI than others. Bagante et al.[22] demonstrated that in a large cohort comprising 1,206 patients, PNI was more prevalent in the PI subtype of ICC than in the MF subtype. This finding was subsequently confirmed by studies involving both smaller and similarly sized cohorts.[52,66] Our data further support these observations, demonstrating an inverse correlation between PNI and the proportion of the MF subtype. This finding indirectly indicates that higher proportions of PI and IG subtypes are associated with higher rates of PNI. These findings are biologically plausible, as PI tumors tend to be more invasive and less well differentiated; therefore, they are more frequently resected with R1 margins. Given that PNI status often correlates with microscopically positive resection margins (R1), this association may also contribute to the observed differences in PNI prevalence between ICC subtypes. Sigel et al.[57] compared small- and large-duct patterns among ICCs and found that the large-duct pattern was associated with a significantly higher PNI prevalence of 71%. Yamashita et al.[70] highlighted that tumor location had a significant impact on the PNI prevalence, with perihilar tumors exhibiting PNI more frequently than peripheral tumors. Similarly, Ebata et al.[30] corroborated this observation; in their comparative analysis of perihilar ECC and ICC, they reported comparable biological behavior, with a remarkably high PNI prevalence of 90.4% among ICC cases. Conversely, Ke et al.[38] reported a lower proportion of PNI-positive patients (<10%) in their cohort of patients with ICC with clinically negative LNM. These findings support the hypothesis that PNI reflects an overall aggressive tumor phenotype associated with LNM and VI, rather than representing an independent prognostic factor (see section below).

In ECC and pancreatic cancer, PNI is a well-established adverse prognostic factor.[14,15] In ICC, however, evidence has been inconsistent. Approximately one-third of studies identified PNI as an independent adverse prognostic factor for OS. Although our meta-analysis supports its overall detrimental impact, the independent impact of PNI on OS remains debatable. Several explanations may account for this inconsistency. First, PNI frequently coexists with other indicators of aggressive tumor biology, such as LNM, VI, and tumor subtypes. The observed prognostic impact of PNI may thus be difficult to disentangle from the underlying stage of disease and, importantly, from intrinsic tumor growth patterns. Jansen et al.[37] demonstrated a significant association of PNI with LNM in ICC—a well-established determinant of poor survival.[80] Similarly, in gastric cancer, Duraker et al.[81] reported that the presence of PNI correlated with tumor volume, depth of invasion, angiogenesis, and lymph node involvement, supporting the concept that PNI reflects an overall aggressive tumor phenotype rather than a stand-alone prognostic factor. Moreover, in contrast to the well-established impact of PNI in ECC, the absence of a consistent prognostic effect in ICC may reflect underlying biological differences between intrahepatic and extrahepatic bile duct tumors.[82] The relationship between tumor growth pattern and histopathological subtype, and the potential impact and prevalence of PNI on prognosis in ICC, is a subject of ongoing research. In particular, differences in tumor morphology and histopathological classification, including MF vs. PI phenotypes and WHO-based small-duct vs. large-duct subtypes, are likely key drivers of both PNI prevalence and clinical outcomes. Large-duct type tumors and PI growth patterns are associated with higher rates of PNI and more aggressive behavior.[34,57] Accordingly, PNI may represent a

surrogate of these adverse biological features rather than an independent determinant of prognosis.

The substantial variability in reported PNI prevalence across studies appears to be multifactorial, reflecting not only biological differences but also methodological inconsistencies in study design and pathological assessment. Although study size and year of publication were identified as relevant moderators, a substantial proportion of the variability in reported PNI prevalence remained unexplained. This is possibly a result of unmeasured differences across studies, particularly the evolution and inconsistency of pathological sampling strategies and diagnostic definitions over time. Most studies did not specify how PNI status was assessed, suggesting that no standardized sampling strategy was applied. In routine practice, many pathology departments examine only a limited number of tumor blocks (*e.g.* two to three regions) rather than performing extensive sampling owing to financial and workload constraints. This approach can lead to imprecise classification of ICC subtypes and may also reduce the sensitivity for detecting PNI. The variability of reported PNI prevalence may be related to the lack of a concise and universally accepted definition of this pathological process. Early definition by Batsakis[83] in 1985—*tumor cell invasion in, around, and through the nerves*—left room for interpretation and was not precise. However, Liebig *et al.*[13] in 2009 suggested a more definite criteria for defining PNI—*finding tumor cells within any of the three layers of the nerve sheath or tumor foci outside of the nerve with involvement of ≥33% of the nerve's circumference are sufficient features for calling PNI*. An adaption of this definition might explain the reduction of PNI prevalence in more recent publications. With regard to sample size, small-study effects should also be considered. Meta-regression demonstrated an inverse association between study size and reported PNI prevalence, indicating that smaller cohorts tended to report higher prevalence estimates than larger cohorts, which may have contributed to the observed heterogeneity. Although the pooled estimate appeared robust in fail-safe N analysis, the presence of significant funnel plot asymmetry indicates that small-study effects may have influenced the results. Nevertheless, leave-one-out sensitivity analyses showed that no individual study exerted a disproportionate influence on the overall pooled prevalence estimate.

Strengths and limitations

The strengths of this study include its large sample size, systematic approach, robust sensitivity analyses, and detailed moderator exploration. Importantly, multivariable meta-regression demonstrated that nearly half of the heterogeneity in prevalence estimates could be explained by study-level characteristics. Limitations must also be acknowledged. The principal limitation of this study is the high degree of heterogeneity observed in the prevalence analysis, which restricts the clinical interpretability of the pooled prevalence estimate. Most included studies were retrospective and thus subject to inherent biases. Most studies did not describe the definition of PNI or specify how PNI assessment and tissue sampling were performed; additionally, reporting of clinicopathological data was not always consistent. HRs for OS were not consistently reported, thereby limiting the number of studies eligible for quantitative synthesis and potentially introducing selection bias. Moreover, variability in multivariable adjustment across studies may affect the comparability of reported effect estimates. The correlation analyses of PNI with other adverse histopathological features were performed at the study level and therefore reflect ecological associations between study-specific prevalence estimates rather than patient-level co-occurrence of these features. Furthermore, our analysis was based predominantly on studies conducted in Asian populations. Although geographic region was not identified as a significant moderator, this predominance of Asian cohorts may still limit the generalizability of the findings to other populations.

Conclusions

PNI is present in approximately one-third of patients with ICC, is associated with poorer clinical outcomes, and clusters with other adverse histopathological features, particularly LNM and VI, underscoring its association with aggressive tumor biology. Although these findings are tempered by substantial heterogeneity, they consistently highlight the adverse impact of PNI. Standardized pathological definitions, together with prospective studies employing uniform assessment and reporting, are needed to better define the prognostic and clinical significance of PNI in ICC.

Affiliations

[1]*Charité – Universitätsmedizin Berlin, Department of Radiology, Berlin, Germany;* [2]*Caritas Klinik – Pankow, Department of Gastroenterology, Berlin, Germany;* [3]*Charité – Universitätsmedizin Berlin, Department of Pathology, Berlin, Germany;* [4]*Charité – Universitätsmedizin Berlin, Department of Surgery CCM / CVK, Berlin, Germany*

Abbreviations

AJCC, American Joint Committee on Cancer; CA19-9, carbohydrate antigen 19-9; df, degrees of freedom; ECC, extrahepatic cholangiocarcinoma; HR, hazard ratio; ICC, intrahepatic cholangiocarcinoma; IDPCC, intraductal papillary carcinoma component; IG, intraductal growth; LNM, lymph node metastasis; LVI, lymphovascular invasion; MF, mass-forming; OS, overall survival; PI, periductal-infiltrating; PNI, perineural invasion; PRISMA, Preferred Reporting Items for Systematic Reviews and Meta-Analyses; RFS, recurrence-free survival; VI, vascular invasion.

Financial support

Open Access funding was enabled and organized by Projekt DEAL. No funding was received.

Conflicts of interest

All authors report that they have no conflict of interest.

Please refer to the accompanying ICMJE disclosure forms for further details.

Authors' contributions

Conceptualization: JK, DG, UF, WS, TAA. Data curation: CWP, CH, TAA, JK. Formal analysis: DG, JK, UF. Investigation: all authors. Methodology: JK, DG. Supervision: UF, WS, DG. Visualization: CWP, JK, CH. Writing – original draft: JK, TAA, UF, DG. Writing – review and editing: all authors.

Data availability

The datasets generated and/or analyzed during the current study are not publicly available because of restrictions related to data ownership, but they are available from the corresponding author on reasonable request.

Declaration of generative AI and AI-assisted technologies in the manuscript preparation process

During the preparation of this work, the authors used ChatGPT (GPT-5.3 series and GPT-5.4 series) for language editing and internal revision. Following the use of these tools, the authors critically reviewed, revised, and validated the content as necessary and assume full responsibility for the final content and conclusions presented in the published article.

Acknowledgements

The authors thank all colleagues and collaborators who contributed valuable discussions and feedback.

Supplementary data

Supplementary data to this article can be found online at https://doi.org/10.1016/j.jhepr.2026.101939.

References

Author names in bold designate shared co-first authorship

[1] Sung H, Ferlay J, Siegel RL, et al. Global cancer statistics 2020: GLOBOCAN estimates of incidence and mortality worldwide for 36 cancers in 185 countries. CA Cancer J Clin 2021;71:209–249.
[2] Banales JM, Cardinale V, Carpino G, et al. Expert consensus document: cholangiocarcinoma: current knowledge and future perspectives consensus statement from the European Network for the Study of Cholangiocarcinoma (ENS-CCA). Nat Rev Gastroenterol Hepatol 2016;13:261–280.
[3] Khan AS, Dageforde LA. Cholangiocarcinoma. Surg Clin North Am 2019;99:315–335.
[4] Yamasaki S. Intrahepatic cholangiocarcinoma: macroscopic type and stage classification. J Hepatobiliary Pancreat Surg 2003;10:288–291.
[5] Jarnagin WR, Fong Y, DeMatteo RP, et al. Staging, resectability, and outcome in 225 patients with hilar cholangiocarcinoma. Ann Surg 2001;234:507–517. ; discussion 517–09.
[6] Endo I, Gonen M, Yopp AC, et al. Intrahepatic cholangiocarcinoma: rising frequency, improved survival, and determinants of outcome after resection. Ann Surg 2008;248:84–96.
[7] Squires MH, Cloyd JM, Dillhoff M, et al. Challenges of surgical management of intrahepatic cholangiocarcinoma. Expert Rev Gastroenterol Hepatol 2018;12:671–681.
[8] Mazzaferro V, Gorgen A, Roayaie S, et al. Liver resection and transplantation for intrahepatic cholangiocarcinoma. J Hepatol 2020;72:364–377.
[9] Chen X, Du J, Huang J, et al. Neoadjuvant and adjuvant therapy in intrahepatic cholangiocarcinoma. J Clin Transl Hepatol 2022;10:553–563.
[10] Panizza B, Warren T. Perineural invasion of head and neck skin cancer: diagnostic and therapeutic implications. Curr Oncol Rep 2013;15:128–133.
[11] Feng FY, Qian Y, Stenmark MH, et al. Perineural invasion predicts increased recurrence, metastasis, and death from prostate cancer following treatment with dose-escalated radiation therapy. Int J Radiat Oncol Biol Phys 2011;81:e361–e367.
[12] Chatterjee D, Katz MH, Rashid A, et al. Perineural and intraneural invasion in posttherapy pancreaticoduodenectomy specimens predicts poor prognosis in patients with pancreatic ductal adenocarcinoma. Am J Surg Pathol 2012;36:409–417.
[13] Liebig C, Ayala G, Wilks JA, et al. Perineural invasion in cancer: a review of the literature. Cancer 2009;115:3379–3391.
[14] Murakami Y, Uemura K, Sudo T, et al. Perineural invasion in extrahepatic cholangiocarcinoma: prognostic impact and treatment strategies. J Gastrointest Surg 2013;17:1429–1439.
[15] Zhou Y, Liu S, Wu L, et al. Survival after surgical resection of distal cholangiocarcinoma: a systematic review and meta-analysis of prognostic factors. Asian J Surg 2017;40:129–138.
[16] Zhang Z, Zhou Y, Hu K, et al. Perineural invasion as a prognostic factor for intrahepatic cholangiocarcinoma after curative resection and a potential indication for postoperative chemotherapy: a retrospective cohort study. BMC Cancer 2020;20:270.
[17] Liberati A, Altman DG, Tetzlaff J, et al. The PRISMA statement for reporting systematic reviews and meta-analyses of studies that evaluate healthcare interventions: explanation and elaboration. BMJ 2009;339:b2700.
[18] Barker TH, Hasanoff S, Aromataris E, et al. The revised JBI critical appraisal tool for the assessment of risk of bias for cohort studies. JBI Evid Synth 2025;23:441–453.
[19] Ahn CS, Hwang S, Lee YJ, et al. Prognostic impact of hepatitis B virus infection in patients with intrahepatic cholangiocarcinoma. ANZ J Surg 2018;88:212–217.
[20] Aishima S, Asayama Y, Taguchi K, et al. The utility of keratin 903 as a new prognostic marker in mass-forming-type intrahepatic cholangiocarcinoma. Mod Pathol 2002;15:1181–1190.
[21] Alaimo L, Lima HA, Moazzam Z, et al. Development and validation of a machine-learning model to predict early recurrence of intrahepatic cholangiocarcinoma. Ann Surg Oncol 2023;30:5406–5415.
[22] Bagante F, Weiss M, Alexandrescu S, et al. Long-term outcomes of patients with intraductal growth sub-type of intrahepatic cholangiocarcinoma. HPB (Oxford) 2018;20:1189–1197.
[23] **Bai S**, **Shi X**, Dai Y, et al. The preoperative scoring system combining neutrophil/lymphocyte ratio and CA19-9 predicts the long-term prognosis of intrahepatic cholangiocarcinoma patients undergoing curative liver resection. BMC Cancer 2024;24:1106.
[24] **Bartsch F**, **Heuft LK**, Baumgart J, et al. Influence of lymphangio (L), vascular (V), and perineural (Pn) invasion on recurrence and survival of resected intrahepatic cholangiocarcinoma. J Clin Med 2021;10:2426.
[25] **Bednarsch J**, **Tan X**, Czigany Z, et al. The presence of small nerve fibers in the tumor microenvironment as predictive biomarker of oncological outcome following partial hepatectomy for intrahepatic cholangiocarcinoma. Cancers 2021;13:3661.
[26] **Chang ME**, **Lei HJ**, Chen MH, et al. Evaluation of prognostic factors and implication of lymph node dissection in intrahepatic cholangiocarcinoma: 10-year experience at a tertiary referral center. J Chin Med Assoc 2017;80:140–146.
[27] Chen IY, Dunne RF, Liao X. Prognostic implications of tumor histology and microenvironment in surgically resected intrahepatic cholangiocarcinoma: a single institutional experience. Virchows Archiv 2025;486:323–332.
[28] Chen TC, Jan YY, Yeh TS. K-ras mutation is strongly associated with perineural invasion and represents an independent prognostic factor of intrahepatic cholangiocarcinoma after hepatectomy. Ann Surg Oncol 2012;19:675–681.
[29] de Franchis V, Petrungaro S, Pizzichini E, et al. Cholangiocarcinoma malignant traits are promoted by Schwann cells through TGFβ signaling in a model of perineural invasion. Cells 2024;13:366.
[30] Ebata T, Kamiya J, Nishio H, et al. The concept of perihilar cholangiocarcinoma is valid. Br J Surg 2009;96:926–934.
[31] Ercolani G, Dazzi A, Giovinazzo F, et al. Intrahepatic, peri-hilar and distal cholangiocarcinoma: three different locations of the same tumor or three different tumors? Eur J Surg Oncol 2015;41:1162–1169.
[32] Fisher SB, Patel SH, Kooby DA, et al. Lymphovascular and perineural invasion as selection criteria for adjuvant therapy in intrahepatic cholangiocarcinoma: a multi-institution analysis. HPB (Oxford) 2012;14:514–522.
[33] Fujita N, Asayama Y, Nishie A, et al. Mass-forming intrahepatic cholangiocarcinoma: enhancement patterns in the arterial phase of dynamic hepatic CT-correlation with clinicopathological findings. Eur Radiol 2017;27:498–506.
[34] Hayashi A, Misumi K, Shibahara J, et al. Distinct clinicopathologic and genetic features of 2 histologic subtypes of intrahepatic cholangiocarcinoma. Am J Surg Pathol 2016;40:1021–1030.
[35] Hirohashi K, Uenishi T, Kubo S, et al. Histologic bile duct invasion by a mass-forming intrahepatic cholangiocarcinoma. J Hepatobiliary Pancreat Surg 2002;9:233–236.
[36] Hu LS, Weiss M, Popescu I, et al. Impact of microvascular invasion on clinical outcomes after curative-intent resection for intrahepatic cholangiocarcinoma. J Surg Oncol 2019;119:21–29.
[37] Jansen R, Moehlendick B, Bartenhagen C, et al. ACGH detects distinct genomic alterations of primary intrahepatic cholangiocarcinomas and matched lymph node metastases and identifies a poor prognosis subclass. Sci Rep 2018;8:10637.
[38] **Ke Q**, **Wang L**, **Lin Z**, et al. Prognostic value of lymph node dissection for intrahepatic cholangiocarcinoma patients with clinically negative lymph node metastasis: a multi-center study from China. Front Oncol 2021;11:585808.
[39] Kubo Y, Aishima S, Tanaka Y, et al. Different expression of glucose transporters in the progression of intrahepatic cholangiocarcinoma. Hum Pathol 2014;45:1610–1617.
[40] Kuroda Y, Aishima S, Taketomi A, et al. 14-3-3σ negatively regulates the cell cycle, and its down-regulation is associated with poor outcome in intrahepatic cholangiocarcinoma. Hum Pathol 2007;38:1014–1022.
[41] Lee CT, Wu TT, Lohse CM, et al. High-mobility group AT-hook 2: an independent marker of poor prognosis in intrahepatic cholangiocarcinoma. Hum Pathol 2014;45:2334–2340.

[42] **Li Q**, **Liu H**, Ma Y, et al. Who benefits from adjuvant chemotherapy? Identification of early recurrence in intrahepatic cholangiocarcinoma patients after curative-intent resection using machine learning algorithms. Front Oncol 2025;15:1594200.
[43] **Li R**, **Chen B**, **Chen Z**, et al. Impact of sarcopenia on the short-term and long-term outcomes of intrahepatic cholangiocarcinoma undergoing hepatectomy: a multi-center study. Eur J Surg Oncol 2024;50:108246.
[44] **Liang KH**, **Yeh TS**, Wu RC, et al. GALNT14 genotype is associated with perineural invasion, lymph node metastasis and overall survival in resected cholangiocarcinoma. Oncol Lett 2017;13:4215–4223.
[45] Liao X, Agostini-Vulaj D, Li RX, et al. Characterization of cholangiocarcinomas with tubulocystic morphology associated with biliary adenofibroma or biliary adenofibroma-like lesions. Mod Pathol 2025:100815.
[46] **Liu Z**, **Luo C**, **Chen X**, et al. Noninvasive prediction of perineural invasion in intrahepatic cholangiocarcinoma by clinicoradiological features and computed tomography radiomics based on interpretable machine learning: a multicenter cohort study. Int J Surg 2024;110:1039–1051.
[47] Ma ZJ, Xiang JX, Weiss M, et al. Long-term survivors after curative-intent resection for intrahepatic cholangiocarcinoma. J Surg Oncol 2024;130: 443–452.
[48] Mano Y, Aishima S, Fukuhara T, et al. Decreased roundabout 1 expression promotes development of intrahepatic cholangiocarcinoma. Hum Pathol 2013;44:2419–2426.
[49] **Misumi K**, **Hayashi A**, Shibahara J, et al. Intrahepatic cholangiocarcinoma frequently shows loss of BAP 1 and PBRM 1 expression, and demonstrates specific clinicopathological and genetic characteristics with BAP 1 loss. Histopathology 2017;70:766–774.
[50] Qi Z, Yuan H, Li Q, et al. An MRI-based fusion model for preoperative prediction of perineural invasion status in patients with intrahepatic cholangiocarcinoma. World J Surg Oncol 2025;23:164.
[51] Raghavan K, Jeffrey RB, Patel BN, et al. MDCT diagnosis of perineural invasion involving the celiac plexus in intrahepatic cholangiocarcinoma: preliminary observations and clinical implications. Am J Roentgenol 2015;205:W578–W584.
[52] Rhee H, Kim MJ, Park YN, et al. A proposal of imaging classification of intrahepatic mass-forming cholangiocarcinoma into ductal and parenchymal types: clinicopathologic significance. Eur Radiol 2019;29:3111–3121.
[53] Sakata K, Yoshizumi T, Izumi T, et al. The role of DNA repair glycosylase OGG1 in intrahepatic cholangiocarcinoma. Anticancer Res 2019;39:3241–3248.
[54] Sarcognato S, Sacchi D, Fabris L, et al. Ferroptosis in intrahepatic cholangiocarcinoma: IDH1(105GGT) single nucleotide polymorphism is associated with its activation and better prognosis. Front Med (Lausanne) 2022;9:886229.
[55] Shinozaki A, Shibahara J, Noda N, et al. Claudin-18 in biliary neoplasms. Its significance in the classification of intrahepatic cholangiocarcinoma. Virchows Arch 2011;459:73–80.
[56] Shirai K, Ebata T, Oda K, et al. Perineural invasion is a prognostic factor in intrahepatic cholangiocarcinoma. World J Surg 2008;32:2395–2402.
[57] Sigel CS, Drill E, Zhou Y, et al. Intrahepatic cholangiocarcinomas have histologically and immunophenotypically distinct small and large duct patterns. Am J Surg Pathol 2018;42:1334–1345.
[58] Tabrizian P, Jibara G, Hechtman JF, et al. Outcomes following resection of intrahepatic cholangiocarcinoma. HPB (Oxford) 2015;17:344–351.
[59] Tajima Y, Kuroki T, Fukuda K, et al. An intraductal papillary component is associated with prolonged survival after hepatic resection for intrahepatic cholangiocarcinoma. Br J Surg 2004;91:99–104.
[60] Takemura Y, Ojima H, Oshima G, et al. Gamma-synuclein is a novel prognostic marker that promotes tumor cell migration in biliary tract carcinoma. Cancer Med 2021;10:5599–5613.
[61] Tan G, Wang WQ, Yuan T, et al. Machine learning prediction of perineural invasion in intrahepatic cholangiocarcinoma. Eur J Surg Oncol 2025;51:110203.
[62] Tanaka M, Shibahara J, Ishikawa S, et al. EVI1 expression is associated with aggressive behavior in intrahepatic cholangiocarcinoma. Virchows Archiv 2019;474:39–46.
[63] Tarchi P, Tabrizian P, Prigoff J, et al. Outcomes of resection for solitary ≤5 cm intrahepatic cholangiocarcinoma. Surgery 2018;163:698–702.
[64] Terashi T, Aishima S, Taguchi K, et al. Decreased expression of osteopontin is related to tumor aggressiveness and clinical outcome of intrahepatic cholangiocarcinoma. Liver Int 2004;24:38–45.
[65] Wang SY, Jiang N, Zeng JP, et al. Characteristic of perineural invasion in hilar cholangiocarcinoma based on whole-mount histologic large sections of liver. Front Oncol 2022;12:855615.
[66] Wei T, Zhang XF, Pawlik TM. ASO author reflections: classification of intrahepatic cholangiocarcinoma into perihilar versus peripheral subtype. Ann Surg Oncol 2024;31:1274–1275.
[67] Wei T, Zhang XF, He J, et al. Prognostic impact of perineural invasion in intrahepatic cholangiocarcinoma: multicentre study. Br J Surg 2022;109: 610–616.
[68] Wu G, Chen X, Luo R, et al. Histopathologic grading of residual tumor predicts survival of intrahepatic cholangiocarcinoma patients treated with neoadjuvant therapy: major pathologic response and its clinical significance. Am J Surg Pathol 2025;49:578–587.
[69] Yamamoto Y, Türkoğlu MA, Aramaki T, et al. Vascularity of Intrahepatic cholangiocarcinoma on computed tomography is predictive of lymph node metastasis. Ann Surg Oncol 2016;23:485–493.
[70] Yamashita YI, Wang H, Kurihara T, et al. Clinical significances of preoperative classification of intrahepatic cholangiocarcinoma: different characteristics of perihilar vs. peripheral ICC. Anticancer Res 2016;36:6563–6569.
[71] Yang XQ, Xu YF, Guo S, et al. Clinical significance of nerve growth factor and tropomyosin-receptor-kinase signaling pathway in intrahepatic cholangiocarcinoma. World J Gastroenterol 2014;20:4076–4084.
[72] Yeh YC, Lei HJ, Chen MH, et al. C-reactive protein (CRP) is a promising diagnostic immunohistochemical marker for intrahepatic cholangiocarcinoma and is associated with better prognosis. Am J Surg Pathol 2017;41:1630–1641.
[73] Yoo T, Park SJ, Han SS, et al. Postoperative CA19-9 change is a useful predictor of intrahepatic cholangiocarcinoma survival following liver resection. Dis Markers 2015;2015:298985.
[74] Yoshikawa D, Ojima H, Iwasaki M, et al. Clinicopathological and prognostic significance of EGFR, VEGF, and HER2 expression in cholangiocarcinoma. Br J Cancer 2008;98:418–425.
[75] **Yuan L**, **Luo X**, Lu X, et al. Comparison of clinicopathological characteristics between cirrhotic and non-cirrhotic patients with intrahepatic cholangiocarcinoma: a large-scale retrospective study. Mol Clin Oncol 2017;7: 615–622.
[76] Zhang H, Huang G, Li Q, et al. Construction and validation of a novel tumor morphology immune inflammatory nutritional score (TIIN score) for intrahepatic cholangiocarcinoma: a multicenter study. BMC Cancer 2024;24:630.
[77] Zhang X, Zhou Y, Wu Z, et al. Double-negative α-fetoprotein and carbohydrate antigen 19-9 predict a good prognosis in intrahepatic cholangiocarcinoma: a propensity score matching analysis. Clin Transl Gastroenterol 2021;12:e00425.
[78] **Zheng Y**, **Huang N**, Kuang S, et al. The clinicopathological significance and relapse predictive role of tumor microenvironment of intrahepatic cholangiocarcinoma after radical surgery. Cancer 2023;129:393–404.
[79] **Zou M**, **Sheng J**, Ruan M, et al. Perineural invasion confers poorer clinical outcomes in patients with T1/T2 intrahepatic cholangiocarcinoma: a single center, retrospective cohort study. J Gastrointest Oncol 2023;14: 2500–2510.
[80] Kolck J, Auer TA, Walter-Rittel T, et al. Prediction of regional lymph node metastasis in intrahepatic cholangiocarcinoma: it's not all about size. Abdom Radiol (NY) 2023;48:3063–3071.
[81] Duraker N, Şişman S, Can G. The significance of perineural invasion as a prognostic factor in patients with gastric carcinoma. Surg Today 2003;33: 95–100.
[82] Spencer K, Pappas L, Baiev I, et al. Molecular profiling and treatment pattern differences between intrahepatic and extrahepatic cholangiocarcinoma. J Natl Cancer Inst 2023;115:870–880.
[83] Batsakis JG. Nerves and neurotropic carcinomas. Ann Otol Rhinol Laryngol 1985;94:426–427.
[84] Bahmad HF, Wegner C, Nuraj J, et al. Perineural Invasion in Breast Cancer: A Comprehensive Review. Cancers 2025;17:1900.

Keywords: Biliary tract cancer; Hepatobiliary malignancy; Liver malignancy; Nerve sheath invasion; Tumor biology; Subtypes; Mass-forming; Periductal-infiltrating; Intraductal growth; Large-duct type; Small-duct type; Biomarker; Prevalence; Curative resection; Outcome; Prognosis; Systematic review; PRISMA; Node-negative disease; Hazard ratio.
Received 12 December 2025; received in revised form 6 June 2026; accepted 23 June 2026; Available online 25 June 2026

Journal of Hepatology, Volume *8*

Supplemental information

Perineural invasion in intrahepatic cholangiocarcinoma: Meta-analysis of prevalence, association with tumor features, and impact on survival

Johannes Kolck, Timo Alexander Auer, Camilla Winther Pedersen, Clarissa Hosse, Jana Ihlow, Felix Krenzien, Wenzel Schöning, Uli Fehrenbach, and Dominik Geisel

Perineural invasion in intrahepatic cholangiocarcinoma: meta-analysis of prevalence, association with tumor features, and impact on survival

Johannes Kolck, Timo Alexander Auer, Camilla Winther Pedersen, Clarissa Hosse, Jana Ihlow, Felix Krenzien, Wenzel Schöning, Uli Fehrenbach, Dominik Geisel

Table of content

Table S1……………………………………………………………………………………2

Table S1

Supplementary Table 1: Risk of bias assessment using JBI Critical Appraisal Checklist ratings for all included cohort studies. The 11-item JBI tool evaluates methodological quality across the following domains: (1) similarity of groups and recruitment from the same population; (2) similarity and consistency of exposure measurement used to classify groups; (3) validity and reliability of exposure measurement; (4) identification of important confounding factors; (5) use of appropriate strategies to deal with confounding; (6) confirmation that participants were free of the outcome at the start of follow-up; (7) validity and reliability of outcome measurement; (8) sufficiency and reporting of follow-up time; (9) completeness of follow-up and description of losses; (10) strategies used to address incomplete follow-up; and (11) appropriateness of statistical analysis. Responses were marked as ✔ (criterion met), ✘ (criterion not met), "? "(unclear), and — (not applicable). An overall risk-of-bias judgement was derived based on the number of unmet/unclear items, with categories defined as low risk (0–1 missing items), moderate risk (2–3 missing items), and high risk (≥4 missing items).

Study	1	2	3	4	5	6	7	8	9	10	11	Missing
Ahn 2018	✔	✔	✔	✔	✔	✔	✔	✔	✔	—	✔	0
Aishima 2002	✔	✔	✔	✔	✔	✔	✔	✔	?	?	✔	2
Alaimo 2023	✔	✔	✔	✔	✔	✔	✔	✔	?	?	✔	2
Bagante 2018	✔	✔	✔	✔	✔	✔	✔	✔	?	?	✔	2
Bai 2024	✔	✔	✔	✔	✔	✔	✔	✔	✘	—	✔	1
Bartsch 2021	✔	✔	✔	✔	✔	✔	✔	✔	?	?	✔	2
Bednarsch 2021	✔	✔	✔	✔	✔	✔	✔	✔	?	?	✔	2
Chang 2017	✔	✔	✔	✔	✔	✔	✔	✔	✔	—	✔	0
Chen 2025	✔	✔	✔	✔	✔	✔	✔	✔	?	?	✔	2
Chen 2000	✔	✔	✔	✔	✔	✔	✔	✔	?	?	✔	2
de Franchis 2024	✔	✔	✔	?	?	—	✔	—	—	—	✔	2
Ebata 2009	✔	✔	✔	✔	✔	✔	✔	✔	?	✘	✔	2
Ercolani 2015	✔	✔	✔	✔	✔	✔	✔	✔	?	✘	✔	2
Fisher 2012	✔	✔	✔	✔	✔	✔	✔	✔	?	✘	✔	2
Fujita 2017	✔	✔	✔	✔	✔	✔	✔	✔	✘	✘	✔	2
Hayashi 2016	✔	✔	✔	✔	✔	✔	✔	✔	✘	✘	✔	2
Hirohashi 2002	✔	✔	✔	✔	✘	✔	✔	✔	?	✘	✔	3
Hu 2019	✔	✔	✔	✔	✔	✔	✔	✔	?	✘	✔	2
Jansen 2018	✔	✔	✔	✔	✘	✔	✔	✔	?	✘	✔	3
Ke 2021	✔	✔	✔	✔	✔	✔	✔	✔	✘	✘	✔	2
Kubo 2014	✔	✔	✔	✔	✘	✔	✔	✔	?	✘	✔	3
Kuroda 2007	✔	✔	✔	✔	✔	✔	✔	✔	?	✘	✔	2
Lee 2014	✔	✔	✔	✔	✔	✔	✔	✔	✘	✘	✔	2
Li 2025	✔	✔	✔	✔	✔	✔	✔	✔	✔	—	✔	0
Li 2024	✔	✔	✔	✔	✔	✔	✔	✔	?	✘	✔	2
Liang 2017	✔	✔	✔	✔	✔	✔	✔	✔	?	✘	✔	2
Liao 2025	✔	✔	✔	✔	✔	✔	✔	✔	?	✘	✔	2
Liu 2024	✔	✔	✔	✔	✔	✔	✔	✔	?	✘	✔	2
Ma 2024	✔	✔	✔	✔	✔	✔	✔	✔	✔	✘	✔	1
Mano 2013	✔	✔	✔	✔	✔	✔	✔	✔	?	✘	✔	2
Misumi 2016	✔	✔	✔	✔	✔	✔	✔	✔	?	✘	✔	2
Qi 2025	✔	✔	✔	✔	✔	✔	✔	—	✔	—	✔	0
Raghavan 2015	✔	✔	✔	✘	✘	✔	✔	—	✔	—	✔	2
Rhee 2019	✔	✔	✔	✘	✘	✔	✔	✔	✔	✘	✔	3
Sakata 2019	✔	✔	✔	✔	✔	✔	✔	✔	?	✘	✔	2
Sarcognato 2022	✔	✔	✔	✔	✔	✔	✔	✔	?	✘	✔	2
Shinozaki 2011	✔	✔	✔	✔	✔	✔	✔	✔	?	✘	✔	2
Shirai 2008	✔	✔	✔	✔	✔	✔	✔	✔	✔	✘	✔	1
